# Phylogeographic analysis of the genus *Platycephalus* along the coastline of the northwestern Pacific inferred by mitochondrial DNA

**DOI:** 10.1186/s12862-019-1477-1

**Published:** 2019-07-31

**Authors:** Jie Cheng, Zhiyang Wang, Na Song, Takashi Yanagimoto, Tianxiang Gao

**Affiliations:** 1 Key Laboratory of Marine Genetics and Breeding (Ocean University of China), Ministry of Education, 5 Yushan Road, Qingdao, 266003 Shandong China; 2 Laboratory for Marine Fisheries Science and Food Production Processes, Pilot National Laboratory for Marine Science and Technology (Qingdao), 1 Wenhai Road, Qingdao, 266237 China; 3 Ocean School, Yantai University, 30 Qingquan Road, Yantai, 264005 Shandong China; 4 Key Laboratory of Mariculture (Ocean University of China), Ministry of Education, 5 Yushan Road, Qingdao, 266003 Shandong China; 5 National Research Institute of Fisheries Science, Japan Fisheries Research and Education Agency, 2-12-4 Fukuura, Kanazawa, Yokohama, Kanagawa 236-8648 Japan; 6 Fishery College, Zhejiang Ocean University, No. 1 Haida South Road, Zhoushan, 316022 Zhejiang China

**Keywords:** *Platycephalus*, Phylogeography, Demographic history, Pleistocene, Northwestern Pacific Ocean

## Abstract

**Background:**

Flathead fishes of the genus *Platycephalus* are economically important demersal fishes that widely inhabit the continental shelves of tropical and temperate sea waters. This genus has a long history of taxonomic revision, and recently four *Platycephalus* species (*Platycephalus* sp. 1, *Platycephalus* sp. 2, *P. indicus,* and *P. cultellatus*) in the northwestern Pacific Ocean (NWP) have been recognized and redescribed. However, many aspects of their systematics and evolutionary history are unclear.

**Results:**

A total of 411 individuals were sampled from 22 different sites across their distributions in the NWP. Three mitochondrial loci were sequenced to clarify the phylogeny and phylogeographic history of the fishes. The results showed significant differentiation of four *Platycephalus* species in the NWP with well-supported clades in which *Platycephalus* sp. 1 and *Platycephalus* sp. 2 were the closest, clustered with *P. cultellatus*, while their genetic relationship with *P. indicus* was the furthest. There were significant genealogical branches corresponding to *P. indicus* but not to other *Platycephalus* lineages. We further examined the phylogeographic patterns of 16 *Platycephalus* sp. 1 populations along the coastlines of China and Japan. A total of 69 haplotypes were obtained, with 23 shared among populations. One dominant haplotypic group, with a modest lineage structure and low levels of haplotype diversity and nucleotide diversity, was observed among *Platycephalus* sp. 1 populations. The demographic history reconstruction suggested a *Platycephalus* sp. 1 population expansion event dating back to the late Pleistocene.

**Conclusions:**

Distributional rang variations may be the crucial factors shaping the genetic relationships of the genus *Platycephalus*. Reproductive schooling and potential egg/larval dispersal ability, coupled with the effects of ocean currents, are responsible for the present phylogeographic pattern of *Platycephalus* sp. 1.

**Electronic supplementary material:**

The online version of this article (10.1186/s12862-019-1477-1) contains supplementary material, which is available to authorized users.

## Background

In marine environments, the phylogeography of organisms may be shaped by complex interactions of extrinsic ecological factors (climatic changes, water temperature, ocean currents, anthropogenic influences, etc.) and intrinsic life-history characteristics (dispersal capacity, pelagic larval duration, etc.) [1, 2]. Moreover, the phylogeography of species can be demonstrated by study of species genetic diversity, population genetic structures and demographic history [1, 3].

During glacial cycles, continental shelves became alternately exposed and submerged under sea water, driving demographic extinction or expansion in numerous marine species [4]. In the Pleistocene glacial period, environmental changes were magnified in the marginal seas of the western Pacific Ocean [5]. With its distinct geographical features of various marginal seas, wide latitudinal range, and complex geological history, the northwestern Pacific (NWP) is an excellent natural region for investigating the genetic consequences of Pleistocene glaciation on population structures and geographical differentiation of marine fish species [6]. The Chinese coastal seas, including the Bohai Sea, Yellow Sea, East China Sea, and South China Sea, are relatively young marginal seas of the western Pacific Ocean. The life-history characteristics of marine fish species, their complex habitats, and seasonally changing current systems in Chinese coastal seas [7], could interact to promote, maintain, or homogenize genetic divergence after glacial periods [8, 9]. Accordingly, marine organisms inhabiting Chinese coastal seas are vital materials for studying the relationship between population genetic structures and geographical distributions [10–12].

Flathead fishes of genus *Platycephalus*, belonging to Family Platycephalidae, Order Scorpaeniformes, are widely distributed in tropical and temperate areas of the Indo-Western Pacific (IWP) and eastern Mediterranean [13–17]. They mainly inhabit estuarine and coastal seas to the edge of the continental shelf. With eyes on the upper surface of flattened heads, they often rest on the sea bottom and engage in ambush hunting. Flatheads have long been economically important fishes in some parts of east Asia and Australia [16]. They were among the top fishes to be commercially targeted by the Australian trawling industry [18]. The taxonomy of flatheads has a long history of confusion; approximately 150 species names have been proposed, but only 77 are considered valid [15–17, 19]. Cryptic species of flatheads have been recognized only recently, mostly in large expanses of the tropical IWP, such as in Australian waters [14, 17, 19, 20]. For example, a phylogenetic analysis of the genus *Platycephalus* with *COI* barcoding by Puckridge et al. [17] demonstrated a high level of genetic diversity of *P. indicus* with eight separated lineages distributed across large expanses of the IWP. The possibility of further cryptic speciation in this and other apparently widely distributed flatheads in the IWP needs to be considered.

There has long been only a single species of *Platycephalus*, *P. indicus,* recorded and studied in the NWP [21, 22]. Recently, newly recognized *Platycephalus* sp. 1 in China and *Platycephalus* sp. 2 in Japan were documented based on morphological characters and DNA barcoding, proving the validity of these species at the genetic level [21–24]. This recognition may coincide with the cryptic speciation of this widely distributed flathead species in the IWP. Meanwhile, *P. cultellatus* and *P. indicus* were also redescribed from the northern South China Sea [22, 25]. All four *Platycephalus* species distributed in the NWP are very similar in morphological and meristic values, with almost all characters overlapping [21, 22, 25]. This similarity leads to frequent misidentification of *Platycephalus* species in the NWP by regional ichthyologists. Given the glacial cycles mentioned above and the recent anthropogenic pressures, the phylogeographic history of the four *Platycephalus* species recorded in the NWP remains unclear. Moreover, no systematic research has been performed to study the population genetics of the newly recognized *Platycephalus* sp. 1, which is the only species widely distributed along Chinese coastal seas.

In the present study, populations of the genus *Platycephalus* were sampled along the coasts of China and Japan, as well as from extra-IWP reference locations. The specimens were identified as four species to investigate the phylogeny and evolutionary history of the genus *Platycephalus*. The phylogenetic relationships were reconstructed with multiple mitochondrial loci. These data were also used to characterize the population structure of the *Platycephalus* sp. 1 throughout its range. The population history of *Platycephalus* sp. 1 was inferred, and the results were used to illuminate how this species responded to the severe climatic fluctuations in the Pleistocene ice ages. Such information may assist in fisheries and ecosystem conservation and investigations of cryptic speciation in the genus *Platycephalus* in the NWP.

## Results

### Sequence variation within the genus *Platycephalus*

**Fig. 1:**
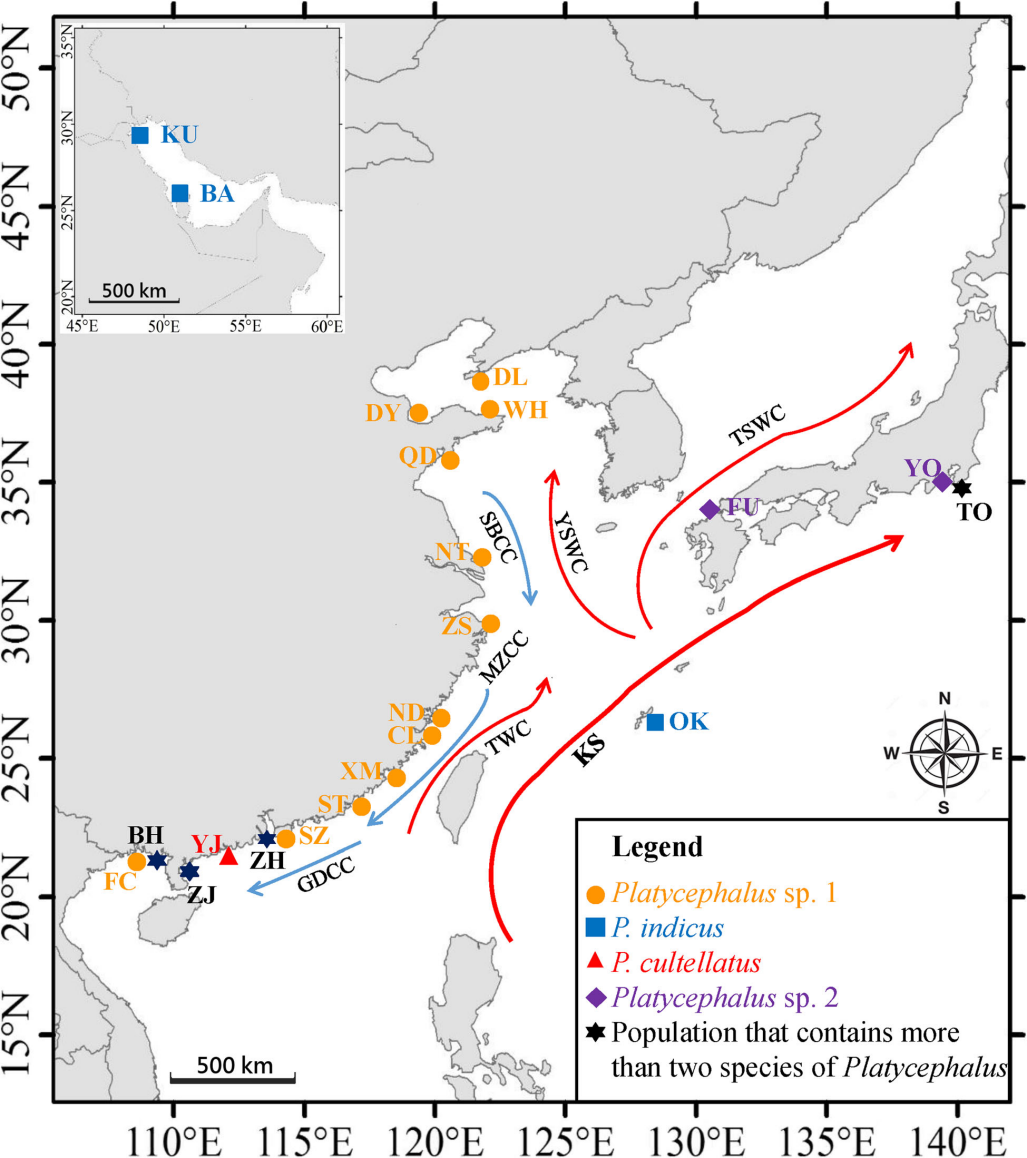
Sample locations for fishes of the genus *Platycephalus* and contemporary marine currents of the NWP. Samples are marked by abbreviations that correspond to those in Table 1 and Additional file 1: Table S1. KS: Kuroshio Current; TWC: Taiwan Warm Current; TSWC: Tsushima Warm Current; YSWC: Yellow Sea Warm Current; SBCC: Subei Coastal Current; MZCC: Minzhe Coastal Current; GDCC: Guangdong Coastal Current. The coastline map was originally made with Natural Earth (http://www.naturalearthdata.com/) and modified with marine currents according to Xu et al. [6]

We obtained 651-bp *COI* sequences and 402-bp Cyt *b* sequences from 151 specimens of 22 populations of the four *Platycephalus* species (Fig. 1 and Additional file 1: Table S1). Comparison of the concatenated *COI* and Cyt *b* sequences revealed 57 distinct haplotypes among the four species, with a haplotype diversity (*h*) of 0.91 ± 0.02. These haplotypes were not shared among species. The numbers of haplotypes observed within each species were 30 for *Platycephalus* sp. 1, 16 for *P. indicus*, six for *P. cultellatus,* and five for *Platycephalus* sp. 2. Moreover, *P. indicus* exhibited the highest level of haplotype diversity (*h* = 0.90 ± 0.03), and *P. cultellatus* exhibited the lowest haplotype diversity (*h* = 0.60 ± 0.15), with *Platycephalus* sp. 1 (*h* = 0.76 ± 0.05) and *Platycephalus* sp. 2 (*h* = 0.71 ± 0.14) in between. The most common haplotype in *Platycephalus* sp. 1 (P1SH1) was observed in 46.6% of the 16 sampled *Platycephalus* sp. 1 populations, and most of the other haplotypes (72.4%) exhibited only one nucleotide difference.

### Phylogenetic relationships of the genus *Platycephalus*

**Fig. 2:**
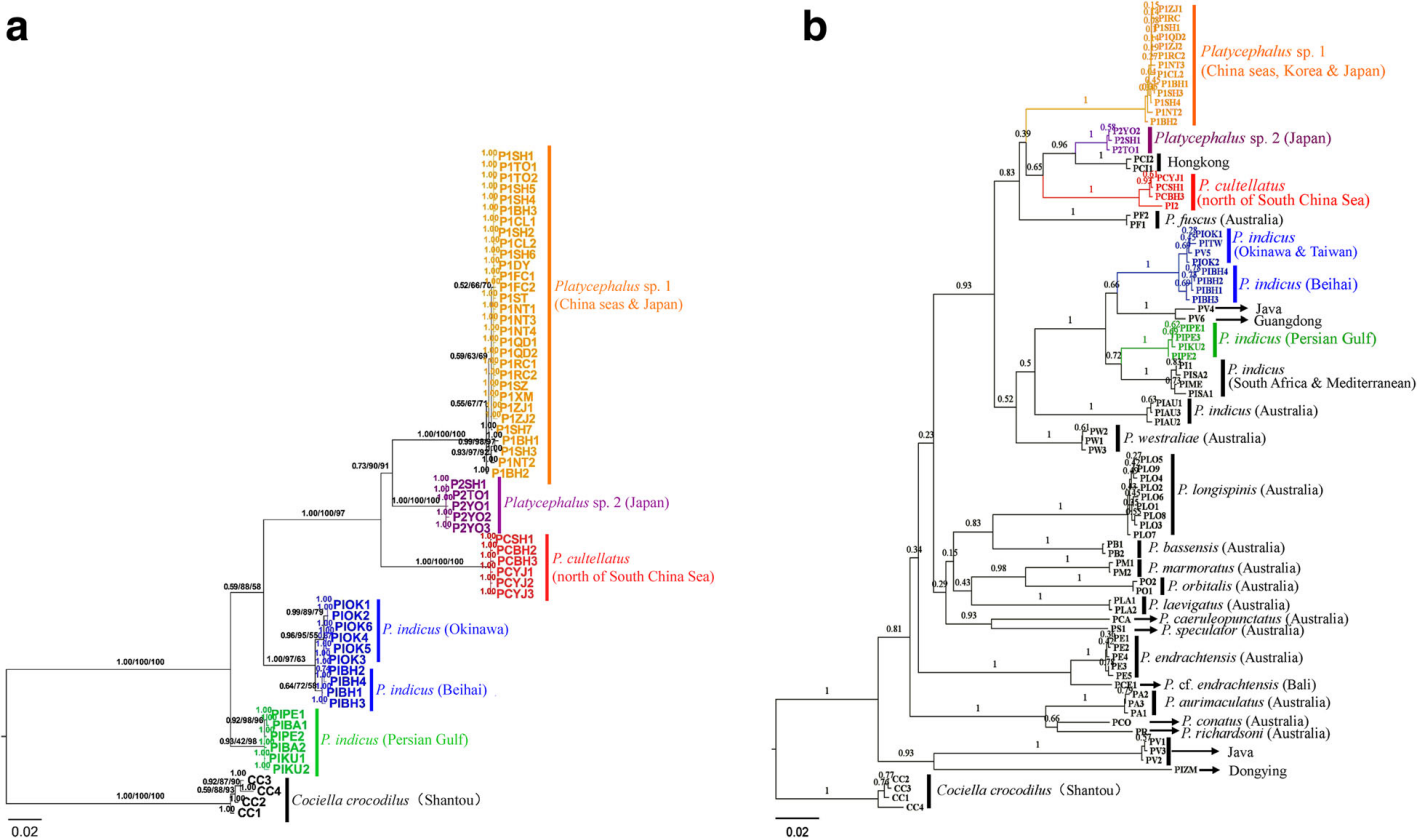
Phylogenetic relationships within the genus *Platycephalus*. **a** Bayesian inference tree based on concatenated *COI* and Cyt *b* sequences from this study. **b** Neighbor-joining tree based on *COI* sequences from this study and from GenBank with the K2P model

The haplotypes from concatenated *COI* and Cyt *b* sequences were used to infer the phylogenetic relationships of the sampled *Platycephalus* species. The concatenated tree topologies from the Bayesian and ML phylogenies were generally congruent (Fig. 2a and Additional file 2: Figure S1). The Bayesian tree (Fig. 2a) revealed a well-resolved phylogeny that included clades for four species. Generally, *Platycephalus* sp. 1 was closest to *Platycephalus* sp. 2, and the two species clustered with *P. cultellatus*, finally the three species clustered with *P. indicus* (Fig. 2a). The topology of the tree was shallow, with no significant genealogical structure regarding sampling locations for *Platycephalus* sp. 1*, Platycephalus* sp. 2, or *P. cultellatus,* respectively, whereas there was an obvious genealogical structure associated with sampling locations among populations of *P. indicus*. A significant lineage structure was distinguished among *P. indicus* of Okinawa, Beihai, and the Persian Gulf (Bahrain and Kuwait).

*COI* sequences from other *Platycephalus* species were utilized to assess the phylogeny and taxonomy of the genus *Platycephalus*; 510-bp *COI* sequences were collected from GenBank and this study (Additional file 3: Table S2). Generally, the phylogenetic relationships among the four NWP *Platycephalus* species considered above were identical to that of the concatenated tree, while there were additional *Platycephalus* species from Australia that were closely related to each other (Fig. 2b and Additional file 4: Figure S2). There were 67 sequences originally named “*P. indicus*” from GenBank; however, haplotype and phylogenetic analyses indicated that 27 of the 67 sequences actually belonged to *Platycephalus* sp. 1 (haplotypes P1SH1, P1SH4, and PIRC), covering samples from Korea, Rongcheng (China), Taiwan (China), and India. Moreover, 12 of the 67 sequences actually belonged to *P. cultellatus* (haplotypes PCSH1 and PCBH3) from the South China Sea, and one sequence belonged to *Platycephalus* sp. 2 from Japan (haplotype P2SH1) (Additional file 3: Table S2).

Lineage structures could be distinguished among geographic locations for populations of *P. indicus*. For example, the *P. indicus* populations from Taiwan (haplotype PITW) and east of Java (haplotype PV5) were more closely related to the population from Okinawa, and they further clustered with populations from the northern South China Sea, while the *P. indicus* samples from Australia, the Persian Gulf, and the Mediterranean were grouped as their own distinct clusters (Fig. 2b).

### Population structure of *P. indicus*

**Fig. 3:**
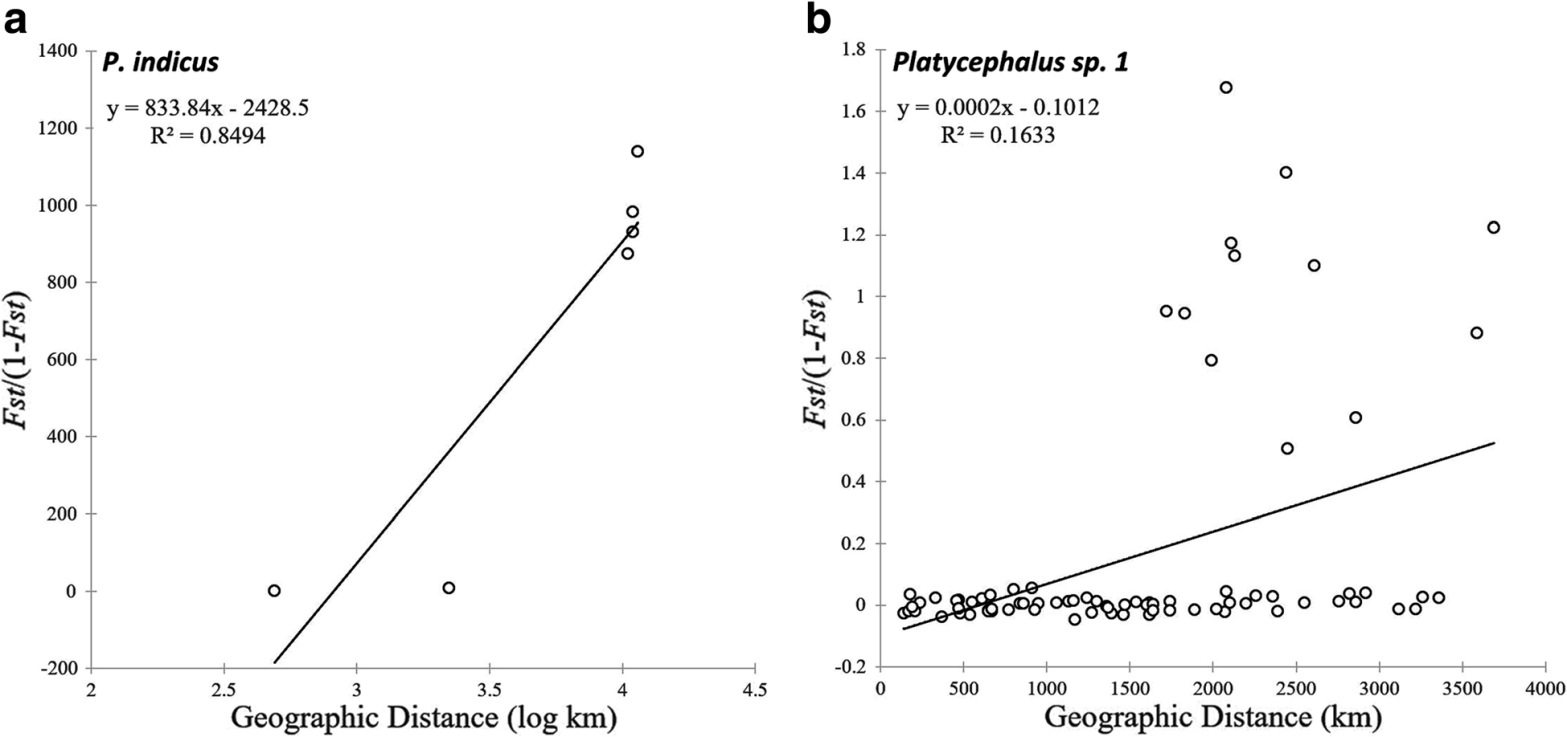
Plots of pairwise estimates of (**a**) *F*_*ST*_/(1 - *F*_*ST*_) vs. log geographic distance between samples of *P. indicus* and (**b**) *F*_*ST*_/(1 - *F*_*ST*_) vs. geographic distance between samples of *Platycephalus* sp. 1. The RMA regression line overlays the scatter plots

The genetic relationships among the four *P. indicus* populations (*n* = 38) was quantified as corrected average pairwise differences, Φ_ST_ values, and AMOVA test results. Except for that between the BA and KU populations from the Persian Gulf, other pairwise values all exhibited large and significant divergence among populations from the South China Sea, Okinawa, and the Persian Gulf, with Φ_ST_ values ranging from 0.7794 to 0.9495 (Table 2A). An AMOVA among three geographically isolated groups of the South China Sea, Okinawa, and the Persian Gulf revealed a high but nonsignificant Φ_CT_ value (Φ_CT_ = 0.93, *P* = 0.1593), with 93.3% of the total genetic variation partitioned among groups. There was a high and significant Φ_ST_ (Φ_ST_ = 0.93, *P* = 0.000) for *P. indicus* populations. Scatter plotting from a Mantel test indicated a significant relationship (*P* = 0.0398) between *F*_*ST*_/(1-*F*_*ST*_) and geographic distance among the four *P. indicus* sample sites (Fig. 3a), indicating isolation by distance, with geographic distance explaining 84.9% of the variation in genetic differentiation for *P. indicus* (*r* = 0.92). The results indicated distance-related genetic differentiation among *P. indicus* populations. Considering the large geographic distance between populations as well as the limited sampling size for *P. indicus*, more samples across their intervening range are required to assess geographic patterns of genetic variation among *P. indicus* populations over their geographical expanse, i.e., whether gradual or with breaks at critical geographic sites.

### Population structure of *Platycephalus* sp. 1

In order to gain more insights into the population and genealogical structure of the newly identified *Platycephalus* sp. 1 in the NWP, we amplified the mtDNA control region of additional specimens (*n* = 348) sampled from 15 Chinese coastal populations and one extra Tokyo Bay population from Japan (Table 1 and Fig. 1). There were 69 haplotypes detected among the 16 populations, and 46 haplotypes were unique and not shared among populations. Among the 23 haplotypes shared among populations, the most common haplotype (SH1) was observed in 156 (44.8%) individuals of the sampled populations, except for Tokyo Bay, Shantou and Zhuhai (Additional file 5: Table S3). Due to the limited number of samples and adjacent collection locations, the samples from Shantou, Shenzhen, Zhuhai, and Zhanjiang were combined and considered one “Guangdong” population in further population structure analyses (Table 1 and Fig. 1). The general haplotype diversity of *Platycephalus* sp. 1 populations was *h* = 0.78 ± 0.02, and the nucleotide diversity was *π* = 0.0041 ± 0.0026. The Nantong population exhibited the highest level of haplotype diversity (*h* = 0.97 ± 0.04), and the Xiamen population exhibited the highest level of nucleotide diversity (*π* = 0.0082 ± 0.0048). The Tokyo Bay population represented both the lowest haplotype diversity (*h* = 0.26 ± 0.12) and the lowest nucleotide diversity (*π* = 0.0012 ± 0.0011). Both the corrected average pairwise differences and the Φ_ST_ values between the Chinese coastal populations were low, nonsignificant and partially negative (Table 2B), which suggested no significant population genetic structure for *Platycephalus* sp. 1 along the coast of China. However, the Tokyo Bay population from Japan exhibited a large and significant divergence from populations in the Chinese coastal seas, with Φ_ST_ values ranging from 0.336 (Xiamen) to 0.626 (Weihai) (*P* < 0.05) (Table 2B).

**Table 1:** Sample information and diversity metrics for *Platycephalus* sp. 1 populations

Population abbreviation	Sample location	Date of sampling	No. of individuals	No. of haplotypes	No. of population-specific haplotypes	No. of polymorphic sites	Haplotype diversity (*h*)	Nucleotide diversity (*π*)
TO	Tokyo Bay	May, 2013	22	4	2	3	0.26 ± 0.12	0.0012 ± 0.0011
DL	Dalian	December, 2012	34	8	2	7	0.66 ± 0.08	0.0024 ± 0.0018
DY	Dongying	July, 2010~October, 2012	32	12	6	14	0.74 ± 0.08	0.0038 ± 0.0025
WH	Weihai	October, 2012	16	7	5	6	0.62 ± 0.14	0.0022 ± 0.0017
QD	Qingdao	October, 2010~October, 2011	40	14	7	13	0.70 ± 0.08	0.0038 ± 0.0025
NT	Nantong	June~December, 2012	15	13	5	13	0.97 ± 0.04	0.0071 ± 0.0043
ZS	Zhoushan	April, 2012	32	13	1	13	0.69 ± 0.09	0.0034 ± 0.0023
ND	Ningde	August, 2012	15	11	3	10	0.93 ± 0.05	0.0046 ± 0.0031
CL	Changle	April, 2013	30	10	1	10	0.60 ± 0.10	0.0028 ± 0.0020
XM	Xiamen	February, 2015	19	12	5	13	0.90 ± 0.06	0.0082 ± 0.0048
ST	Shantou	December, 2015	5	(Guangdong) 14	3	(Guangdong) 17	(Guangdong) 0.91 ± 0.04	(Guangdong) 0.0065 ± 0.0039
SZ	Shenzhen	December, 2014	3		0			
ZH	Zhuhai	Januarary, 2016	2		1			
ZJ	Zhanjiang	December, 2014	15		2			
BH	Beihai	June, 2012~December, 2014	37	11	2	10	0.74 ± 0.07	0.0034 ± 0.0023
FC	Fangchenggang	November, 2014	31	10	1	9	0.74 ± 0.06	0.0030 ± 0.0021
Total			348	139	46	138	0.78 ± 0.02	0.0041 ± 0.0026

Due to the limited number of samples and adjacent collection locations, the samples from Shantou, Shenzhen, Zhuhai, and Zhanjiang were combined and considered as one “Guangdong” population in further population structure analyses

**Table 2:** Corrected average pairwise differences (above diagonal), Φ_***ST***_ values (below diagonal) between populations (abbreviation as in Table 1) and average number of pairwise differences within populations on the diagonal

(A)													
	KO	BH	BA	KU									
KO	10.24	24.58**	123.98**	124.71**									
BH	0.78**	6.01	118.12**	118.38**									
BA	0.93**	0.95**	9.14	−0.41									
KU	0.94**	0.95**	−0.05	6.93									
(B)													
	TO	DL	DY	WH	QD	NT	ZS	ND	CL	XM	GD	BH	FC
TO	**0.54**	1.24*	1.41*	1.24*	1.52*	1.51*	1.10*	0.92*	1.16*	1.02*	1.13*	1.07*	1.27*
DL	0.58*	1.09	0.01	0.02	0.02	0.05	0.00	− 0.01	0.01*	− 0.06	− 0.05	− 0.02	−0.02
DY	0.52*	0.01	1.77	− 0.02	0.02	0.09	0.01	−0.01	0.02	0.09	0.01	0.04	0.04
WH	0.63*	0.02	−0.02	1.03	−0.03	− 0.07	−0.02	0.03	−0.03	− 0.07	−0.02	0.06	0.05
QD	0.53*	0.02	0.01	−0.03	1.76	−0.00	0.04	0.01	0.02	−0.02	−0.04	0.02	0.02
NT	0.49*	0.05	0.05	−0.03	0.01	3.27	0.04	0.09	−0.02	0.06	0.02	0.03	0.02
ZS	0.49*	0.00	0.01	−0.02	0.02	0.03	1.57	−0.06	−0.02	− 0.01	−0.02	0.01	0.01
ND	0.44*	−0.03	0.00	0.02	0.01	0.03	−0.03	2.14	−0.05	−0.10	− 0.03	−0.04	− 0.04
CL	0.54*	0.01	0.01	−0.03	0.01	0.01	−0.02	−0.02	1.31	−0.07	− 0.05	−0.01	0.00
XM	0.34*	−0.02	0.04	−0.03	0.00	0.01	0.00	−0.04	−0.02	3.77	−0.04	− 0.14	−0.08
GD	0.38*	−0.02	0.01	−0.02	−0.01	0.01	−0.00	0.02	−0.02	− 0.01	2.98	− 0.04	−0.04
BH	0.47*	−0.01	0.03	0.04	0.01	0.03	0.01	−0.02	−0.01	− 0.05	−0.01	1.59	−0.01
FC	0.55*	−0.01	0.02	0.04	0.01	0.03	0.00	−0.02	0.00	−0.03	−0.02	− 0.01	1.39

The statistics are based on mitochondrial control region sequences. (A) for *P. indicus* populations; (B) for *Platycephalus* sp. 1 populations. *indicates significance *P* < 0.05 and **indicates significance *P* < 0.01 which are both highlighted in bold

One-group AMOVA revealed a low but significant Φ_ST_ value (Table 3A, Φ_ST_ = 0.08, *P* = 0.0000), which indicated that there was only a modest population structure among populations, with 91.6% of the total genetic variation apportioned within *Platycephalus* sp. 1 populations. When we took the Tokyo Bay population as one group and the Chinese coastal sea populations as another group, there was a significant genetic structure difference between groups (Table 3B, Φ_CT_ = 0.38, *P* = 0.044), with 37.9% of total genetic variation appointed between groups. A total of 61.2% of the total genetic variation was appointed within populations. Moreover, when we further split the Chinese coastal sea populations into the Bohai and Yellow Sea group, the East China Sea group, and the South China Sea group based on potential geographic isolation features, there was a lower, but significant Φ_CT_ value (Table 3C, Φ_CT_ = 0.11, *P* = 0.0078), with only 11.1% of total genetic variation appointed among groups. The constructed group with the Tokyo Bay population splitting off was identified as the optimal group due to the highest significant Φ_CT_ value (Table 3). Therefore, there was great genetic divergence between the Tokyo Bay population and Chinese coastal populations. Scatter plotting from a Mantel test showed no significant relationship (*P* > 0.05) between *F*_*ST*_/(1-*F*_*ST*_) and the geographic distance among the *Platycephalus* sp. 1 populations (Fig. 3b), indicating no isolation by distance, with geographic distance explaining only 16.3% of the variation in genetic differentiation for *Platycephalus* sp. 1 (*r* = 0.40).

**Table 3:** Analysis of molecular variation (AMOVA) of the *Platycephalus* sp. 1 populations based on mitochondrial control region fragments with different group breaks

Groups	Source of variation	Variance components	Percentage of variation	Φ Statistics	*P*
(A) One group	Among populations	0.08	8.4	Φ_ST_ = 0.08	0.0000
	Within populations	0.87	91.6	–	–
(B) Two groups: Pacific coast of Japan, Chinese coastal seas	Among groups	0.39	37.9	Φ_CT_ = 0.38	0.0440
	Among populations within groups	0.01	0.9	Φ_SC_ = 0.01	0.0078
	Within populations	0.63	61.2	Φ_ST_ = 0.38	0.0000
(C) Four groups: Pacific coast of Japan, Bohai and Yellow Sea, East China Sea, South China Sea	Among groups	0.11	11.1	Φ_CT_ = 0.11	0.0078
	Among populations within groups	−0.01	−0.2	Φ_SC_ = -0.01	0.7410
	Within populations	0.87	89.2	Φ_ST_ = 0.11	0.0000

(A) All *Platycephalus* sp. 1 populations were calculated as a whole group; (B) *Platycephalus* sp. 1 populations were split into two groups: Pacific coast of Japan group – TO, and Chinese coastal seas group – all other populations; (C) *Platycephalus* sp. 1 populations were split into four groups: Pacific coast of Japan group - TO, Bohai and Yellow Sea group – DL, DY, WH, QD, NT, East China Sea group – ZS, ND, CL, XM, South China Sea group – GD, BH, FC

**Fig. 4:**
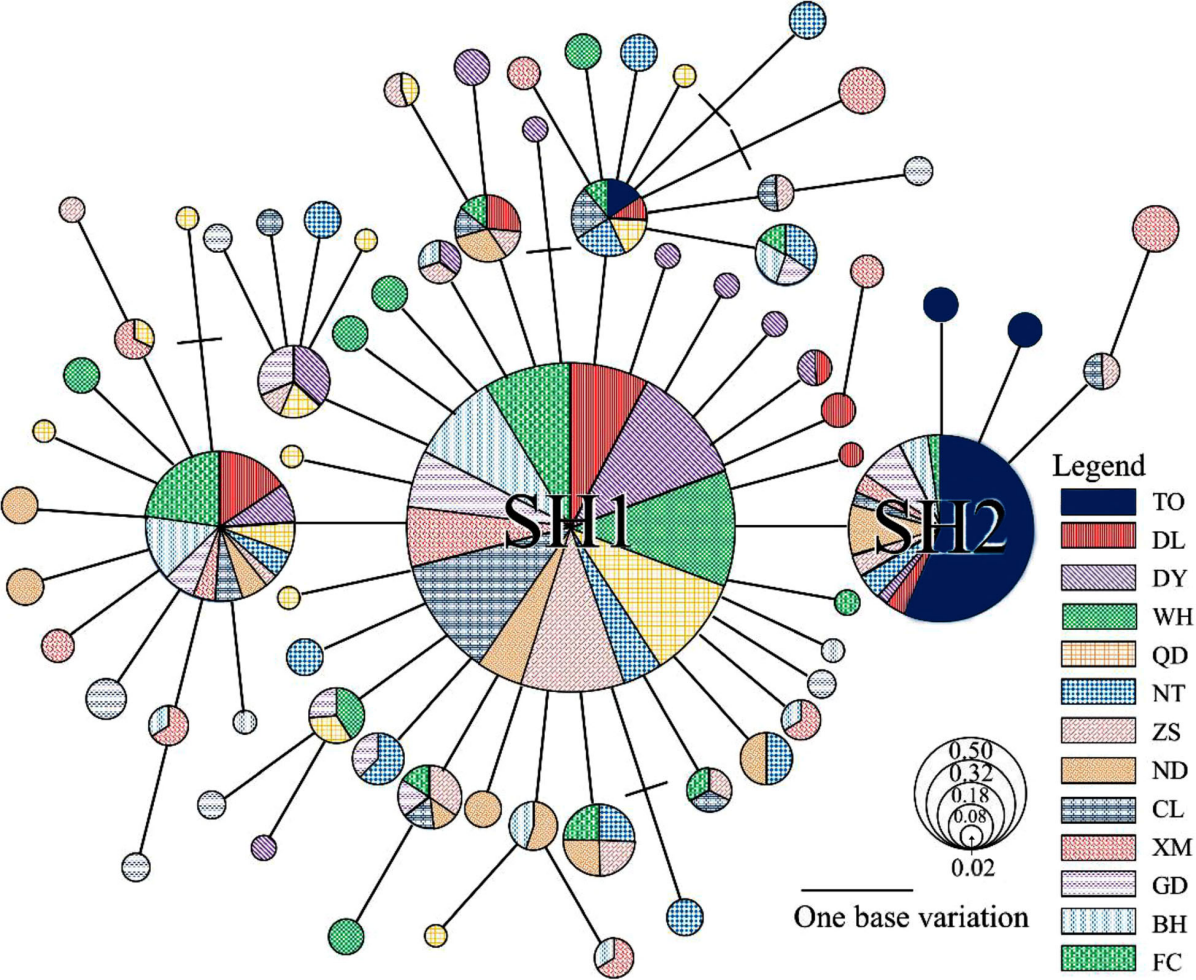
Reduced median network showing the genetic relationships among the control region haplotypes of *Platycephalus* sp. 1 populations. The sizes of the circles represent the sum of haplotype frequencies from all populations. Mutational steps are indicated as bars for the number of substitutions separating two haplotypes. SH1 was the most common haplotype among the Chinese coastal populations, and SH2 was the most common haplotype in the Tokyo Bay population

The genetic relationship among *Platycephalus* sp. 1 populations also was demonstrated by the haplotype distribution network (Fig. 4), which was without clear geographic pattern. The haplotype median-joining network exhibited relatively shallow and single star-shaped polytomies with one frequent central haplotype (SH1) surrounded by 31 other low-frequency haplotypes. The haplotype SH1 contained individuals from all populations except for the Tokyo Bay population, which mainly exhibited the SH2 haplotype (19 of 20 individuals). This structure indicated that there was no obvious genealogical structure among *Platycephalus* sp. 1 populations, and that a population expansion event may have occurred during evolution.

### Demographic history of *Platycephalus* sp. 1

**Fig. 5:**
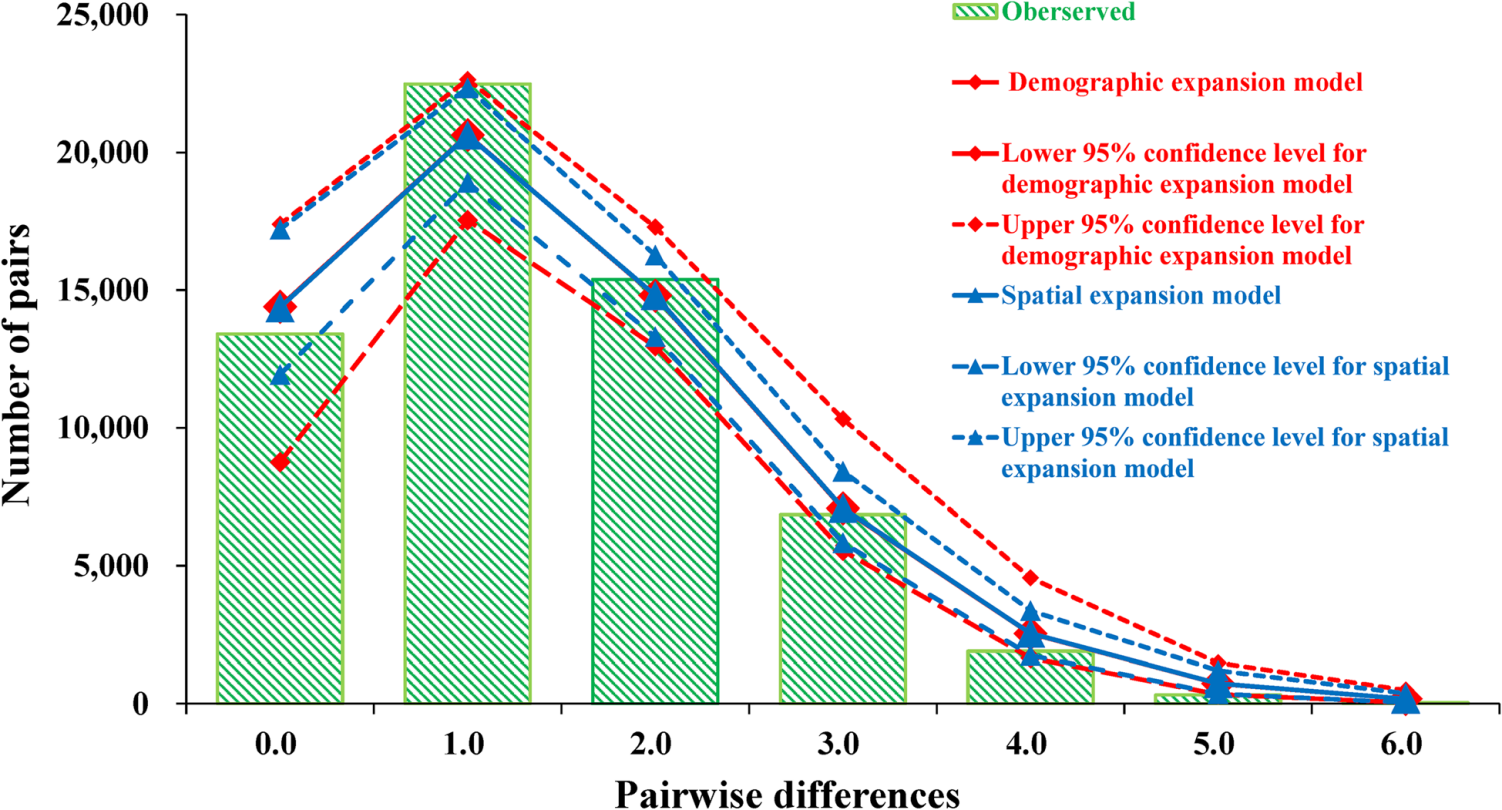
Observed pairwise differences (bars) and the expected mismatch distributions under the sudden expansion model (solid line) of the *Platycephalus* sp. 1 control region haplotypes

A test of the demographic history of *Platycephalus* sp. 1 populations yielded negative and significant results for both Tajima’s *D* (*D* = − 2.29, *P* = 0.0000) and *F*_*s*_ (*F*_*s*_ = − 28.59, *P* = 0.0000) statistics, which indicated a recent population expansion event. Moreover, the sequence mismatch distribution analysis illustrated a unimodal, smooth distribution pattern, which did not differ significantly from the pure demographic and spatial expansion model with a sum-of-squares deviation of 0.0015. This result suggested a typical expansion event in *Platycephalus* sp. 1 populations (Fig. 5). According to the *Ƭ* values (1.42 and 1.43 for each model), as well as the approximate nucleotide substitution rate (3.0–10.0% / Myr) [10, 26], the demographic expansion of *Platycephalus* sp. 1 populations along the coastlines of the NWP could be dated to approximately 30,800 to 103,400 years ago.

**Fig. 6:**
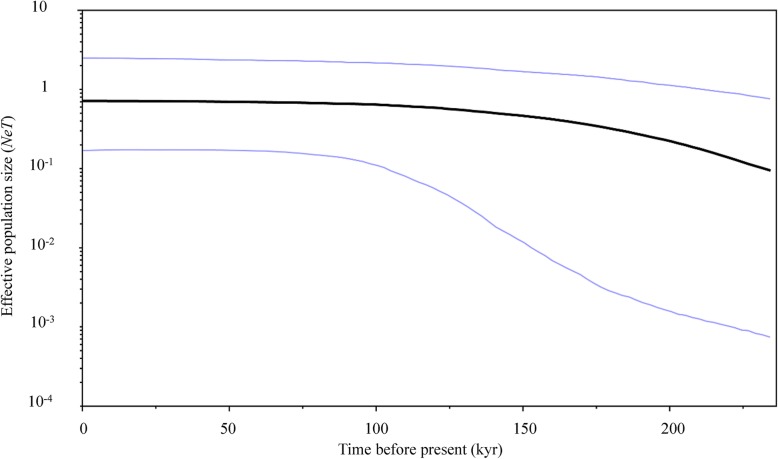
Bayesian skyline plots for *Platycephalus* sp. 1, showing inferred effective population size as a function of time before present. The black line represents median estimate and the light lines are the upper and lower 95% highest posterior density (HPD) limits

The Bayesian skyline plot also revealed a continuous and gentle increase in the effective population size of *Platycephalus* sp. 1 (Fig. 6), the minimum of which could be dated to approximately 23,000 to 75,000 years ago, towards the end of the Pleistocene ice age. This result was consistent with the inferred process of historical expansion of *Platycephalus* sp. 1 populations, as suggested by the significant negative *D* and *F*_*s*_ statistics, as well as the pattern of unimodal mismatch distributions.

## Discussion

In addition to their center of diversity in Australia [15–17, 19], four *Platycephalus* species (*Platycephalus* sp. 1, *Platycephalus* sp. 2, *P. indicus,* and *P. cultellatus*) have recently been recognized and redescribed in the NWP [21–23, 25]. This study aimed to reconstruct the phylogeny and infer the phylogeographic history of the genus *Platycephalus* within the NWP. Phylogenetic analysis revealed significant differentiation among four *Platycephalus* species in the NWP. Variation of mtDNA sequences showed significant genealogical structure differences among *P. indicus* populations across a large expanse*,* but this variation was not observed among populations of the other three species. There was genetic homogeneity among most *Platycephalus* sp. 1 populations along the coast of China, while significant differentiation was observed between populations of China and Japan. The inferred demographic history of *Platycephalus* sp. 1 suggested a population expansion event tracing back to the late Pleistocene.

### Morphology of the genus *Platycephalus* in the NWP

Previously, only a single species, *P. indicus,* was recognized in the NWP, while *Platycephalus* sp. 1 and *Platycephalus* sp. 2 were only recently distinguished from *P. indicus* in China and Japan, with strong evidence for species validity at the genetic level [21–23, 27]. All four *Platycephalus* species recognized in the NWP have overlapping or similar morphological metrics, which resulted in frequent species misidentification, as shown by the few *Platycephalus* sequences in GenBank having been misidentified as *P. indicus* (Additional file 3: Table S2). Based on reports from our group and other studies, we summarized the recently described morphological characteristics of the four *Platycephalus* species along the Chinese coast and Japan, listed in Additional file 6: Table S4. All four species are characterized by 13 normal dorsal- and anal-fin rays, caudal fins with horizontal blackish bands, a scaled interorbital and occipital region, large caniniform teeth absent on the upper jaw, and a finger-like interopercular flap (Additional file 6: Table S4) [21–23, 25].

Among the four species upon which we focus, *P. indicus* is the only species with a single, anterior, small isolated spine on the first dorsal fin and a yellow marking in the middle of the caudal fin, while the other three species normally have two small isolated spines interior to the first dorsal fin and no yellow color on the caudal fin [21–23, 25]. The number of gill rakers and pored lateral line scales can also distinguish the species (Additional file 6: Table S4). The morphological information coupled with DNA barcoding can effectively aid in explicit species taxonomy and avoid misidentification and erroneous distributional records within *Platycephalus* in the NWP.

### Phylogeny of the genus *Platycephalus* in the NWP

The phylogenetic relationships revealed distinct genetic structural differences among the sampled *Platycephalus* species from the NWP (Fig. 2). *Platycephalus* sp. 1 and *Platycephalus* sp. 2 were the most closely related, and these two species clustered with *P. cultellatus*, while the genetic relationship between *P. indicus* and the former three species was the furthest. The distributions of sampled *Platycephalus* species along the Chinese coast were sympatric or parapatric. *Platycephalus* sp. 1 was widely distributed along the Chinese coast, ranging from the Bohai Sea (Dalian) to the South China Sea (Beihai), whereas *Platycephalus* sp. 2 was geographically restricted to the coastal seas of Japan. This finding was consistent with those of reports from Japanese ichthyologists [23, 24, 27] and may be due to their specific thermal adaptation or geographic isolation by large expanses and ocean currents. Meanwhile, the distributions of *P. indicus* and *P. cultellatus* in China were confined to only the northern South China Sea. The abundant distribution of *Platycephalus* sp. 1 and the infrequent presence of *P. indicus* and *P. cultellatus* along the Chinese coast sharply contrasted, which suggested that the latter two species might prefer warmer water. The phylogeny was very shallow, with no clear phylogeographic patterns for *Platycephalus* sp. 1, *Platycephalus* sp. 2, and *P. cultellatus* populations in the NWP. However, a significant lineage structure related to geographical distribution was observed for *P. indicus* populations of Okinawa, Beihai, and the Persian Gulf (Fig. 2 and 3a). This structuring was probably because of obstacles that isolated the respective *P. indicus* populations, such as a large geographical expanse between the Persian Gulf and the other two populations (Fig. 3a), and the strong Kuroshio Current which isolates the Okinawa and Beihai populations (Fig. 1). These geographic and current barriers may have driven *P. indicus* populations to become taxonomically distinct as subspecies or even species. This inference coincided with the results of a previous study [17] in which eight divergent *P. indicus* lineages were designated, including *P. indicus* (China/Korea), *P. indicus* (Japan), *P.* cf. *indicus* (Hongkong), and *P. indicus* (Persian Gulf), which may indicate cryptic speciation. Regarding results of the Puckridge et al. study [17], the newly described *Platycephalus* sp. 1 and *Platycephalus* sp. 2 may correspond to the *P. indicus* (China/Korea) and *P. indicus* (Japan) lineages, respectively. Analysis of more samples across the intervening range is warranted to assess whether there might be gradual variation in *P. indicus* over a geographical expanse.

Geographic boundaries formed during the Pleistocene ice age have also played an important role in species dispersion. The genus *Platycephalus* is widely distributed in tropical and temperate areas of the IWP. This distribution suggests that the lineage could have originated in the tropical region, then dispersed north and became isolated in the NWP during glacial cycles. During the last glacial maximum (LGM), various refugia emerged, and geographic marine obstacles contributed to genetic isolation, which drove the differentiation that resulted in lineage structure divergence related to geographic distribution. For example, *P. cultellatus* was sampled only in the northern South China Sea, whose glacial refuge was the central South China Sea during the LGM. After the LGM, seasonal changes in the coastal currents in the northern South China Sea and the long developmental period of pelagic larvae contributed to the dispersion of the species.

In summary, distributional range variations, probably resulting from the divergence of thermal adaptation and the natural history effects of glaciation, may be the crucial drivers that shaped the genetic relationships of *Platycephalus* species in the NWP. Intrinsic life-history divergence may also have contributed to speciation processes, and hence further investigation into the life-history characteristics of these species is warranted.

### Pleistocene glaciation and patterns of demographic history

Climatic condition during the Pleistocene ice age had dramatic effects on species distributions and abundance in the NWP [10, 28, 29]. Eurythermic species may have occupied various refugia during the glacial periods, which contributed to within-species divergence, while noneurythermic species may have been confined in limited refugia resulting in genetic homogeneity. However, the diverse life histories of marine species and the fluctuating environments (currents, sea level, water temperature, etc.) may have changed the phylogeographical scenario of fishes after their expansion from various Pleistocene refugia. The sequence mismatch distribution pattern of *Platycephalus* sp. 1 populations (Fig. 5) was unimodal and fully consistent with a population expansion scenario. *Platycephalus* sp. 1 populations also displayed a genetic pattern typical of a recent population expansion event, showing a single common haplotype (SH1) across most of its distribution, as well as a shallow star-shaped haplotype network (Fig. 4). *Platycephalus* sp. 1 was widespread along the China coastal seas, but during the LGM, the sea level fell approximately 120 to 140 m [30]. The sea level decline resulted in the exposure of the Yellow Sea and the Bohai Sea, the East China Sea was drawn back to the Okinawa Trough [31], and the South China Sea was separated from the Indian Ocean as a semi-closed sea [32]. Consequently, under the challenging environmental conditions in the glacial periods, *Platycephalus* sp. 1 may have experienced a reduction in population size over large parts of its distribution and survived in various isolated glacial refugia. The geographic isolation of these refugia contributed to genetic divergence among populations, quite possibly with adaptation to local conditions. During the interglacial stage, sea level and temperature rose, which may have contributed to the subsequent dispersal and demographic expansion of *Platycephalus* sp. 1 into the surrounding seas, such as the Yellow Sea, East China Sea, and South China Sea [33–35]. This interpretation also has been suggested for other marine species from this area [36, 37]. However, the data of the present study indicated only a modest population genetic structure, with one dominant haplotype and low levels of haplotype diversity and nucleotide diversity in *Platycephalus* sp. 1. Noting their high fecundity and habit of reproductive schooling, fluctuating oceanographic conditions would have contributed to a tendency for “sweepstake reproduction” for *Platycephalus* sp. 1 populations. In addition, heavy fishery pressure on demographically small lineages also could contribute to genetic homogeneity, causing the SH1 haplotype to become the most frequent haplotype within *Platycephalus* sp. 1 populations. Screenings of nuclear markers are warranted for further investigation of this hypothesis.

### Effects of ocean currents and pelagic larval duration on population structure

Marine fishes generally present low genetic differentiation across geographic regions because of their high dispersal potential with planktonic egg, larval, juvenile, and adult stages. This dispersal could be accompanied by the absence of physical barriers isolating the species among ocean basins or adjacent marginal seas [38–40]. In this study, genetic homogeneity was observed among most of the *Platycephalus* sp. 1 populations along the Chinese coasts, while gene flow was limited only between Tokyo Bay samples and those from China (most of the Φ_ST_ > 0.3, *P* < 0.05) (Table 2B). *Platycephalus* has pelagic larvae that can live in the coastal seas before settling to the seabed as juveniles [41]. The larval duration period is not clear for most *Platycephalus* species. However, larvae of *Platycephalus fuscus* have been found in sea water up to two months after their breeding season [42]. Accordingly, high egg, larvae, or juvenile dispersal potential could be present in *Platycephalus* sp. 1 as well, and this might be an important factor promoting gene flow and homogeneity of populations across large geographic distances.

Marine current dynamics also affect species dispersal. The NWP has complex oceanic current systems along its continental margins, and seasonal current circulations are mostly driven by monsoon winds, for example, leading to water exchanges between the East China Sea and South China Sea through the Taiwan Strait (Fig. 1). Planktonic *Platycephalus* eggs or larvae could be transported northward during summer from the South China Sea to the East China Sea and the Yellow Sea by the Taiwan Warm Current and the Yellow Sea Warm Current [43]. On the other hand, with the winter monsoon winds, coastal waters streaming southward through the Taiwan Strait could drive extensive gene flow among populations along the China coastal seas [44–46]. With these strong marine currents, eggs, larvae, or juveniles of *Platycephalus* sp. 1 can travel long distances despite their limited adult dispersal capabilities. Several other fish species from Chinese seas, such as *Liza affinis* and *Sillago japonica*, with pelagic early-life stages, also could be mixed by ocean currents and present genetic homogeneity among Chinese coastal populations [47, 48]. Moreover, the Tokyo Bay population, which is partially blocked from Chinese coastal populations, showed low but significant genetic divergence from *Platycephalus* sp. 1 populations of the Chinese coast. The Tokyo Bay population apparently receives limited recruitment from the Yellow Sea and the East China Sea, likely due to the isolation by the Japanese landmass as well as the strong Kuroshio Current and Tsushima Warm Current (Fig. 1). This coincides with the long-term climatological events that may have created a barrier between populations of the Japanese and Chinese seas.

## Conclusion

The present study reconstructed the phylogeny and inferred the evolutionary history of the genus *Platycephalus* from the NWP. Mitochondrial DNA sequence analysis demonstrated significant differentiation among the four *Platycephalus* species sampled in the NWP. The genealogical structure was shallow for three *Platycephalus* species, but subclades were detected within *P. indicus*. There was low genetic diversity and homogeneity in *Platycephalus* sp. 1 populations in the Chinese coastal seas. The demographic history of *Platycephalus* sp. 1 suggested a population expansion event dating to the late Pleistocene. Reproductive schooling and high larval dispersal coupled with the complex ocean currents are likely responsible for the present phylogeographic pattern of *Platycephalus* sp. 1. When managing *Platycephalus* fisheries, information that we developed can contribute to maintaining sustainable fisheries, improving ecosystem conservation, and investigating cryptic organismal evolution.

## Methods

### Sample collection

Specimens of the genus *Platycephalus* were collected from 22 locations (Table 1, Additional file 1: Table S1, and Fig. 1): *Platycephalus* sp. 1 were sampled from 15 sites along the Chinese coast and one site from Tokyo Bay, Japan. *Platycephalus* sp. 2 were collected from three sites in Japan. *P. cultellatus* were sampled from four sites in the northern South China Sea, and *P. indicus* were collected from Okinawa, Beihai, and the Persian Gulf (Bahrain and Kuwait). The samples were collected by trawl net in offshore waters from local fishermen or from fish market of local fishing ground, thus ensuring that the samples collected were representative of the local populations. Detailed information about the locations and numbers of specimens collected is listed in Additional file 1: Table S1. All individuals were identified on the basis of morphological characteristics [21, 22, 25], and a piece of muscle was taken from each individual and preserved in 95% ethanol for DNA extraction.

### DNA extraction, PCR and sequencing analysis

Genomic DNA was extracted from muscle tissue by a standard proteinase K digestion and phenol-chloroform extraction method [49]. PCR amplifications were performed for three mitochondrial DNA (mtDNA) fragments: the control region (*CR*), cytochrome *b* (Cyt *b*), and cytochrome c oxidase I (*COI*). PCR was carried out in 25-μl reaction volumes with the primers listed in Additional file 7: Table S5. The PCR-amplified products were gel-separated, purified, sequenced with both primers, and analyzed on an ABI 3730 automated sequencer (Applied Biosystems, Waltham, MA, USA).

Sequence concatenations and file format conversions were implemented in Sequence Matrix [50]. Sequences were proofread, assembled, and aligned using DNASTAR software (DNASTAR, Inc., Madison, WI, USA). Haplotype identifications were determined in DnaSP [51]. Molecular diversity indices, such as the number of haplotypes, haplotype diversity (*h*), and nucleotide diversity (*π*), were obtained in ARLEQUIN [52].

### Partition scheme and model selection

Various partition methods were employed to find the best-fit combination of substitution model parameters for the phylogenetic analysis. The best-fitting nucleotide substitution model was determined using the Bayesian approach implemented in jModelTest [53] for *CR* and in PartitionFinder 2 [54] for *COI* and Cyt *b* sequences. Protein-encoding sequences of *COI* and Cyt *b* were partitioned based on three codon positions, and the best codon substitution model was determined. To select the optimal model parameters and the best phylogenetic tree, all partition schemes were compared with the likelihood ratio test [55] for the maximum likelihood (ML) trees and Bayes factors (BFs) for the Bayesian trees. BFs were computed with a Stepping Stone analysis [56, 57] in MrBayes [58]; the analysis included two MCMC runs with five chains and 100 steps of 1,000,000 generations each, sampling every 1,000 generations, and was stopped when it reached a mean standard deviation between split frequencies of less than 0.01.

### Phylogeny reconstruction for the genus *Platycephalus*

To construct phylogenies based on the mtDNA sequences, six individuals were selected from each population of the sampled *Platycephalus* species*.* If the sampled specimens were fewer than six at a certain location, then all individuals were included. For phylogenetic analysis, 1) concatenated *COI* and Cyt *b* sequences of 151 specimens from populations of the four *Platycephalus* species were analyzed, and 2) homologous *COI* regions for other *Platycephalus* species were searched for using BLAST and downloaded from GenBank (https://www.ncbi.nlm.nih.gov/), excised and included in the combined *COI* phylogeny. The species and their *COI*-sequence GenBank accession numbers are listed in Additional file 3: Table S2. Based on the best-fit model estimated above, the ML tree was constructed with IQ-TERR [59], the Bayesian tree was constructed with MrBayes [58], and the neighbor-joining (NJ) tree was constructed with MEGA 6 [60] using the Kimura two-parameter (K2P) model [61] with 1,000 bootstrap replications. Five specimens of *Cociella crocodile* were used as an outgroup.

### Population genetic structure analysis

Population structures based on the *CR* were analyzed for *P. indicus* and *Platycephalus* sp. 1, but not for *P. cultellatus* and *Platycephalus* sp. 1 due to their limited sample sizes. Pairwise population differences within *P. indicus* and *Platycephalus* sp. 1 populations were estimated with Φ_ST,_ and corrected average pairwise differences were calculated in ARLEQUIN. The statistical significance of these parameters was tested as table-wide significance (*P* < 0.05) with 10,000 permutations. Population structure was evaluated by a hierarchical analysis of molecular variance (AMOVA) [62] using 10,000 permutations in ARLEQUIN to examine the significant variation among groups of genetically similar populations. To evaluate hypothesized patterns of spatial genetic structures, three independent hierarchical AMOVAs were implemented across *Platycephalus* sp. 1 populations. One group was designated with all *Platycephalus* sp. 1 populations. Two groups (Pacific coast of Japan group - TO and Chinese coastal seas group) and four groups (Pacific coast of Japan group – TO; the Bohai and Yellow Sea group – DL, DY, WH, QD, NT; the East China Sea group - ZS, ND, CL, XM; and the South China Sea group – GD, BH, FC) were designated based on potential geographic isolation factors, such as the Tsushima Strait, Yangtze River estuary, and Taiwan Strait (Fig. 1). Moreover, independent hierarchical AMOVAs were implemented across *P. indicus* populations; three groups (the South China Sea group; the Okinawa group; the Persian Gulf group – KU, BA) were designated based on their geographic distance. Genetic split between populations was identified by finding the largest significant values of Φ_CT_ among population groups. Haplotype networks were generally better than phylogenetic trees to describe population relationships [1]. The median joining haplotype network was estimated using Network [63], which was used for constructing large networks post-processed with parsimony [64].

To test for isolation by distance, pairwise values of *F*_*ST*_/(1-*F*_*ST*_) were plotted against geographical distance between sample sites of *Platycephalus* sp. 1 (one-dimensional stepping-stone model), while pairwise values of *F*_*ST*_/(1-*F*_*ST*_) were plotted against the logarithm of distance between sample sites of *P. indicus* (two-dimensional stepping-stone model) considering the wide distribution range of *P. indicus*. The strength and significance of the relationship between genetic distances and geographic distances was assessed using reduced major axis (RMA) regression and Mantel tests using the software ARLEQUIN.

### Demographic history of *Platycephalus* sp. 1

The historical demographic pattern was examined for *Platycephalus* sp. 1 populations. The neutrality mutation test was calculated using Tajima’s *D* [65] and Fu’s *F*_*S*_ [66], with significance tested using 1,000 replicates in ARLEQUIN. Historical demographic expansions also were tested with the mismatch distributions of all pairwise differences in ARLEQUIN for pure demographic [67] or spatial [68] expansions. Raggedness indices were estimated to evaluate the DNA sequence mismatch distributions of the respective populations [69], and the observed frequencies were compared to model predictions based on 10,000 permutations. If the observations were not significantly different from model predictions, the time of expansion was estimated by the equation *Ƭ* = 2 μt*,* where *μ* is the mutation rate [67].

BEAST v.1.7.4 was employed to estimate the changes in effective population size (*N*_e_) across time and to create Bayesian skyline plots [70]. The unique haplotypes of total samples were run for 100,000,000 generations with a constant skyline model of 15 groups. The strict molecular clock model was the prior distribution, with an initial value of 0.06 and a range from 0.03 to 0.10 [10, 26]. To test convergence, trace plots were inspected in Tracer v1.6 [71], and the effective sample sizes (ESSs) were confirmed to exceed 200.

## Additional files

Additional file 1: **Table S1.** Sample information and diversity metrics of the genus *Platycephalus* in the NWP. (DOCX 26 kb)

Additional file 2: **Figure S1.** Phylogenetic relationships of the genus *Platycephalus* based on concatenated *COI* and Cyt *b* sequences. Partitioned maximum likelihood tree and codon maximum likelihood tree are on top and bottom, respectively. (TIF 1372 kb)

Additional file 3: **Table S2.** *COI* sequences of the *Platycephalus* from GenBank. (DOCX 44 kb)

Additional file 4: **Figure S2.** Phylogenetic relationships of the genus *Platycephalus* based on *COI* sequences from this study and from GenBank. Bayesian inference tree, partitioned maximum likelihood tree, and codon maximum likelihood tree are from left to right. (TIF 1953 kb)

Additional file 5: **Table S3.** Distribution of haplotypes shared among populations of the *Platycephalus* sp. 1. (DOCX 17 kb)

Additional file 6: **Table S4.** Comparison of selected morphological characters of four *Platycephalus* species from Chinese coastal seas and Pacific coast of Japan, as found in bibliography. (DOCX 20 kb)

Additional file 7: **Table S5.** Primers information of mitochondrial DNA for the *Platycephalus. (DOCX 19 kb)*

## Data Availability

All relevant data are within the paper and its additional files. We have submitted the mitochondrial DNA sequences to GenBank with accession numbers MH615133-MH615480, MH615481-MH615630, MH615631–615781.

## Abbreviations

*COI*: Cytochrome c oxidase I
*CR*: Control region
Cyt *b*: Cytochrome *b*
*h*: Haplotype diversity
IWP: Indo-Western Pacific
K2P: Kimura two-parameter
LGM: Last glacial maximum
ML: Maximum likelihood
mtDNA: Mitochondrial DNA
NWP: Northwestern Pacific
PCR: Polymerase chain reaction
*π*: Nucleotide diversity
