# Supplementary material for: Phylogeographic analysis of the genus Platycephalus along the coastline of the northwestern Pacific inferred by mitochondrial DNA
Source: BMC Evol Biol. 2019 Jul 31;19:159. doi: 10.1186/s12862-019-1477-1 (PMC6670200; doi:10.1186/s12862-019-1477-1)
Supplement: Supplementary file 1 — Table S1. Sample information and diversity metrics of the genus Platycephalus in the NWP. (DOCX 26 kb) [file 12862_2019_1477_MOESM1_ESM.docx]

Table S1. Sample information and diversity metrics of the genus *Platycephalus* in the NWP.

|  | Population abbreviation | Sample location | Date of sampling | No. of individuals | No. of haplotypes | No. of population-specific haplotypes | No. of polymorphic sites | Haplotype diversity(*h*) | Nucleotide  diversity(*π*) |
| --- | --- | --- | --- | --- | --- | --- | --- | --- | --- |
| *Platycephalus* sp.1 | TO | Tokyo Bay | May, 2013 | 22 | 4 | 2 | 3 | 0.26±0.12 | 0.0012±0.0011 |
|  | DL | Dalian | December, 2012 | 34 | 8 | 2 | 7 | 0.66±0.08 | 0.0024±0.0018 |
|  | DY | Dongying | July, 2010~October, 2012 | 32 | 12 | 6 | 14 | 0.74±0.08 | 0.0038±0.0025 |
|  | WH | Weihai | October, 2012 | 16 | 7 | 5 | 6 | 0.62±0.14 | 0.0022±0.0017 |
|  | QD | Qingdao | October, 2010~ October, 2011 | 40 | 14 | 7 | 13 | 0.70±0.08 | 0.0038±0.0025 |
|  | NT | Nantong | June~December, 2012 | 15 | 13 | 5 | 13 | 0.97±0.04 | 0.0071±0.0043 |
|  | ZS | Zhoushan | April, 2012 | 32 | 13 | 1 | 13 | 0.69±0.09 | 0.0034±0.0023 |
|  | ND | Ningde | August, 2012 | 15 | 11 | 3 | 10 | 0.93±0.05 | 0.0046±0.0031 |
|  | CL | Changle | April, 2013 | 30 | 10 | 1 | 10 | 0.60±0.10 | 0.0028±0.0020 |
|  | XM | Xiamen | February, 2015 | 19 | 12 | 5 | 13 | 0.90±0.06 | 0.0082±0.0048 |
|  | ST | Shantou | December, 2015 | 5 | Guangdong  14 | 3 | Guangdong  17 | Guangdong  0.91±0.04 | Guangdong  0.0065±0.0039 |
|  | SZ | Shenzhen | December, 2014 | 3 |  | 0 |  |  |  |
|  | ZH | Zhuhai | Januarary, 2016 | 2 |  | 1 |  |  |  |
|  | ZJ | Zhanjiang | December, 2014 | 15 |  | 2 |  |  |  |
|  | BH | Beihai | June, 2012~December, 2014 | 37 | 11 | 2 | 10 | 0.74±0.07 | 0.0034±0.0023 |
|  | FC | Fangchenggang | November, 2014 | 31 | 10 | 1 | 9 | 0.74±0.06 | 0.0030±0.0021 |
| *P. indicus* | OK | Okinawa | August, 2012 | 6 | 5 | 6 | 19 | - | 0.0081±0.0051 |
|  | BH | Beihai | November, 2015 | 17 | 11 | 12 | 29 | - | 0.0109±0.0059 |
|  | BA | Bahrain | November, 2014 | 7 | 7 | 6 | 21 | - | 0.0165±0.0097 |
|  | KU | Kuwait | July, 2011 | 8 | 8 | 7 | 16 | - | 0.0062±0.0038 |
| *P. cultellatus* | ZH | Zhuhai | January, 2016 | 1 | 1 | 1 | - | - | - |
|  | YJ | Yangjiang | November, 2012~April, 2016 | 48 | 36 | 33 | 62 | 0.94±0.03 | 0.0104±0.0057 |
|  | ZJ | Zhanjiang | December, 2014 | 1 | 1 | 1 | - | - | - |
|  | BH | Beihai | June, 2012~September, 2016 | 30 | 26 | 23 | 62 | 0.99±0.01 | 0.0142±0.0077 |
| *Platycephalus.* sp.2 | TO | Tokyo Bay | May, 2013 | 2 | - | - | - | - | - |
|  | FU | Fukuoka | September, 2012 | 4 | - | - | - | - | - |
|  | YO | Yokosuka | October, 2012 | 5 | - | - | - | - | - |
