# Supplementary figures and images for: Phylogeographic analysis of the genus Platycephalus along the coastline of the northwestern Pacific inferred by mitochondrial DNA
Source: BMC Evol Biol. 2019 Jul 31;19:159. doi: 10.1186/s12862-019-1477-1 (PMC6670200; doi:10.1186/s12862-019-1477-1)

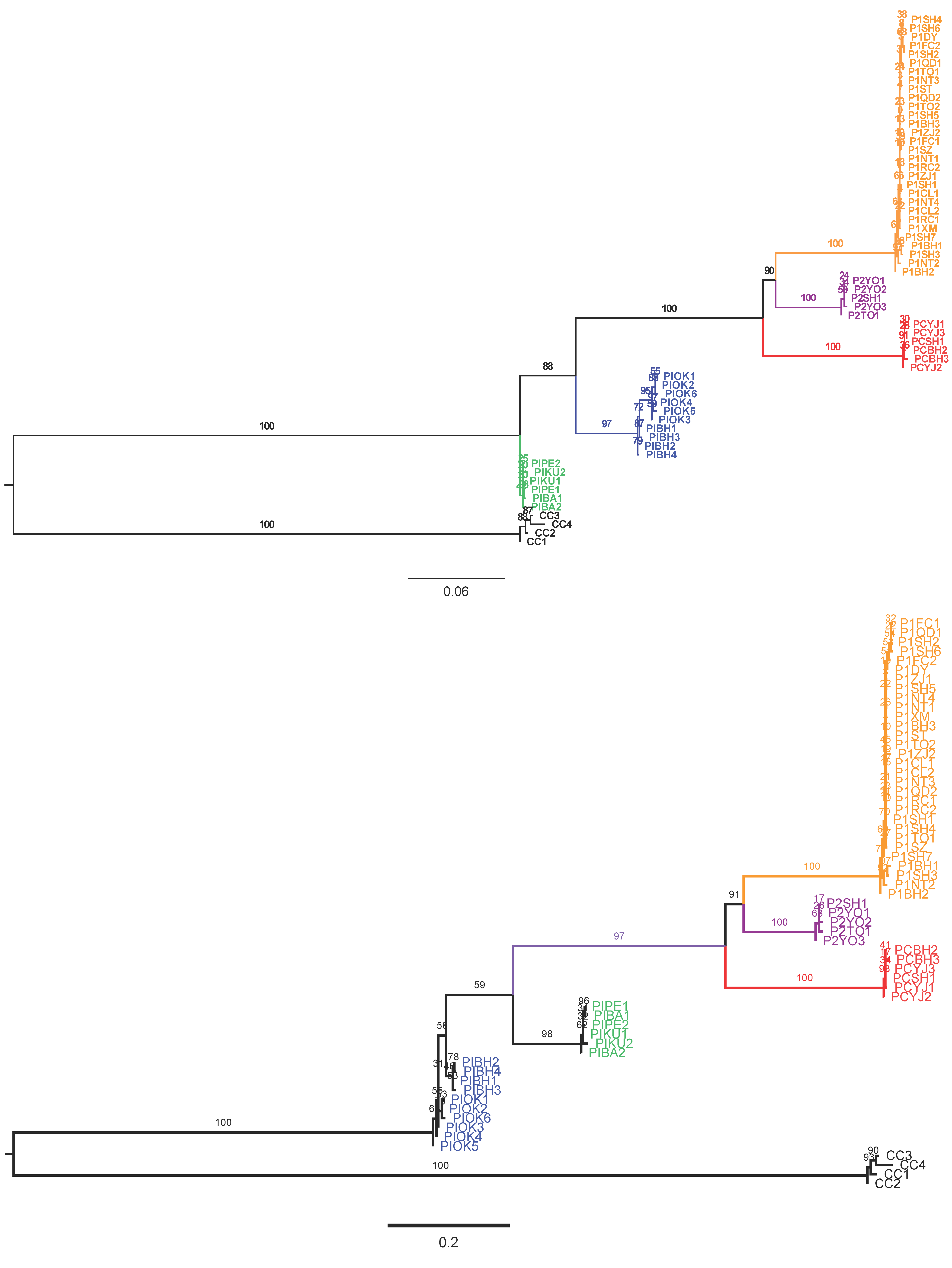

Supplement: Supplementary file 2 — Figure S1. Phylogenetic relationships of the genus Platycephalus based on concatenated COI and Cyt b sequences. Partitioned maximum likelihood tree and codon maximum likelihood tree are on top and bottom, respectively. (TIF 1372 kb) [file 12862_2019_1477_MOESM2_ESM.tif]

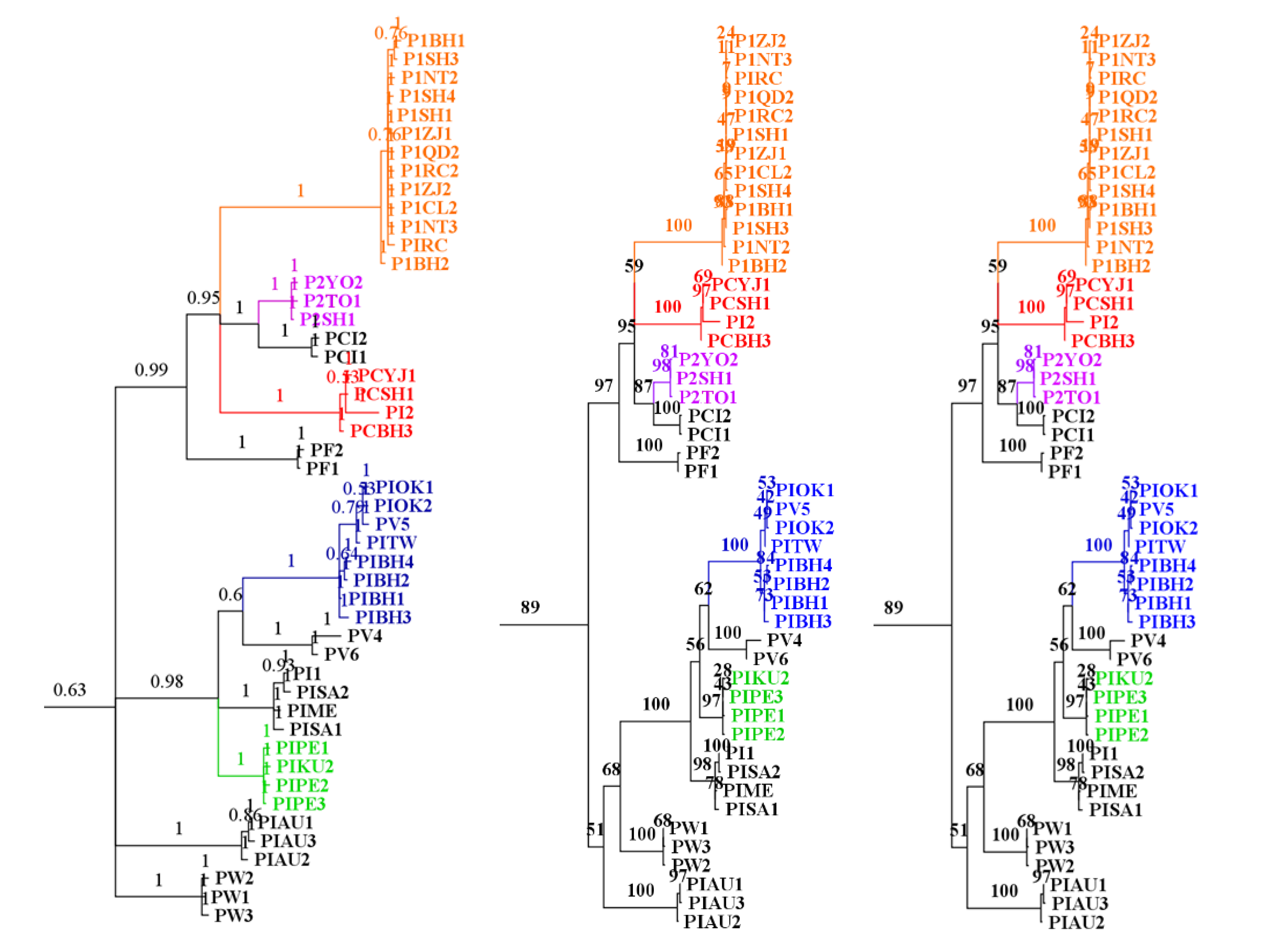

Supplement: Supplementary file 4 — Figure S2. Phylogenetic relationships of the genus Platycephalus based on COI sequences from this study and from GenBank. Bayesian inference tree, partitioned maximum likelihood tree, and codon maximum likelihood tree are from left to right. (TIF 1953 kb) [file 12862_2019_1477_MOESM4_ESM.tif]
