# Supplementary material for: Phylogeographic analysis of the genus Platycephalus along the coastline of the northwestern Pacific inferred by mitochondrial DNA
Source: BMC Evol Biol. 2019 Jul 31;19:159. doi: 10.1186/s12862-019-1477-1 (PMC6670200; doi:10.1186/s12862-019-1477-1)
Supplement: Supplementary file 3 — Table S2. COI sequences of the Platycephalus from GenBank. (DOCX 44 kb) [file 12862_2019_1477_MOESM3_ESM.docx]

Table S2. *COI* sequences of the *Platycephalus* from GenBank.

| No. | GenBank number | Name of haplotype | Scientific name | Speculated sample location |  | Research remark |
| --- | --- | --- | --- | --- | --- | --- |
| 1 | JX488255 | PA1 | *P. aurimaculatus* | Southeast Australia |  |  |
| 2 | JX488155 | PA2 | *P. aurimaculatus* | South Australia |  |  |
| 3 | DQ108005 | PA3 | *P. aurimaculatus* | South Australia |  |  |
| 4 | DQ107991 | PB1 | *P. bassensis* | Southeast Australia |  |  |
| 5 | JX488216 | PB1 | *P. bassensis* | Southeast Australia |  |  |
| 6 | JX488246 | PB1 | *P. bassensis* | Southeast Australia |  |  |
| 7 | JX488169 | PB2 | *P. bassensis* | Southeast Australia |  |  |
| 8 | DQ107996 | PCA | *P. caeruleopunctatus* | Southeast Australia |  |  |
| 9 | DQ107992 | PCA | *P. caeruleopunctatus* | Southeast Australia |  |  |
| 10 | DQ107993 | PCA | *P. caeruleopunctatus* | Southeast Australia |  |  |
| 11 | DQ107994 | PCA | *P. caeruleopunctatus* | Southeast Australia |  |  |
| 12 | DQ107995 | PCA | *P. caeruleopunctatus* | Southeast Australia |  |  |
| 13 | JX488183 | PCO | *P. conatus* | South Australia |  |  |
| 14 | DQ107998 | PE1 | *P. endrachtensis* | Southeast Australia |  |  |
| 15 | DQ108000 | PE1 | *P. endrachtensis* | Southeast Australia |  |  |
| 16 | JX488223 | PE1 | *P. endrachtensis* | Northeast Australia |  |  |
| 17 | JX488274 | PE1 | *P. endrachtensis* | Northeast Australia |  |  |
| 18 | JX488282 | PE1 | *P. endrachtensis* | Northeast Australia |  |  |
| 19 | JX488230 | PE2 | *P. endrachtensis* | Northeast Australia |  |  |
| 20 | DQ108001 | PE3 | *P. endrachtensis* | Southeast Australia |  |  |
| 21 | DQ107999 | PE4 | *P. endrachtensis* | Southeast Australia |  |  |
| 22 | DQ107990 | PE5 | *P. endrachtensis* | Southeast Australia |  |  |
| 23 | JX488179 | PCE1 | *P.* cf. *endrachtensis* | Bali |  |  |
| 24 | JX488221 | PCE1 | *P. cf. endrachtensis* | Bali |  |  |
| 25 | JX488235 | PCE1 | *P. cf. endrachtensis* | Bali |  |  |
| 26 | JX488279 | PCE1 | *P. cf. endrachtensis* | Bali |  |  |
| 27 | JX488285 | PCE1 | *P. cf. endrachtensis* | Central Java |  |  |
| 28 | DQ107974 | PF1 | *P. fuscus* | Southeast Australia |  |  |
| 29 | DQ107987 | PF1 | *P. fuscus* | Southeast Australia |  |  |
| 30 | DQ107988 | PF1 | *P. fuscus* | Southeast Australia |  |  |
| 31 | DQ107989 | PF2 | *P. fuscus* | Southeast Australia |  |  |
| 32 | KU943399 | PITW | *P. indicus* | Taiwan |  | Clustered with *P. indicus* of Okinawa |
| 33 | KU943400 | PITW | *P. indicus* | Taiwan |  | Clustered with *P. indicus* of Okinawa |
| 34 | JN885883 | PIZM | *P. indicus* | Zhimai River, Dongying |  | Not *Platycephalus* |
| 35 | DQ885038 | PI1 | *P. indicus* | — |  |  |
| 36 | DQ885039 | PI1 | *P. indicus* | — |  |  |
| 37 | JF494161 | PI1 | *P. indicus* | Japan |  | Clustered with *P. indicus* of South Africa and Mediterranean |
| 38 | JX488231 | PI1 | *P. indicus* | South Africa |  |  |
| 39 | JX488245 | PI1 | *P. indicus* | South Africa |  |  |
| 40 | JX488281 | PI2 | *P. indicus* | — |  |  |
| 41 | KR861547 | PIME | *P. indicus* | Southeast Mediterranean Sea |  |  |
| 42 | JX488177 | PISA1 | *P. indicus* | South Africa |  |  |
| 43 | JX488174 | PISA2 | *P. indicus* | South Africa |  |  |
| 44 | DQ107979 | PIAU2 | *P. indicus* | Australia |  |  |
| 45 | JX488261 | PIAU1 | *P. cf. indicus* | Northeast Australia |  |  |
| 46 | DQ107976 | PIAU1 | *P. indicus* | Australia |  |  |
| 47 | DQ107977 | PIAU1 | *P. indicus* | Australia |  |  |
| 48 | DQ107978 | PIAU1 | *P. indicus* | Australia |  |  |
| 49 | DQ107975 | PIAU3 | *P. indicus* | Australia |  |  |
| 50 | HQ149899 | PIPE1 | *P. indicus* | Northeast Persian Gulf |  |  |
| 51 | HQ149900 | PIPE2 | *P. indicus* | Northeast Persian Gulf |  |  |
| 52 | HQ149901 | PIPE3 | *P. indicus* | Northeast Persian Gulf |  |  |
| 53 | HQ149902 | PIPE3 | *P. indicus* | Northeast Persian Gulf |  |  |
| 54 | JX972212 | PIBH2 | *P. indicus* | Beihai, China |  |  |
| 55 | EU595226 | PCSH1 | *P. indicus* | South China Sea |  | *P. cultellatus* |
| 56 | EU595227 | PCSH1 | *P. indicus* | South China Sea |  | *P. cultellatus* |
| 57 | EU595228 | PCSH1 | *P. indicus* | South China Sea |  | *P. cultellatus* |
| 58 | EU595229 | PCSH1 | *P. indicus* | South China Sea |  | *P. cultellatus* |
| 59 | FJ238009 | PCSH1 | *P. indicus* | South China Sea |  | *P. cultellatus* |
| 60 | FJ238010 | PCSH1 | *P. indicus* | South China Sea |  | *P. cultellatus* |
| 61 | FJ238011 | PCSH1 | *P. indicus* | South China Sea |  | *P. cultellatus* |
| 62 | KP112375 | PCSH1 | *P. indicus* | Central China |  | *P. cultellatus* |
| 63 | KP112377 | PCSH1 | *P. indicus* | Central China |  | *P. cultellatus* |
| 64 | KP112378 | PCSH1 | *P. indicus* | Central China |  | *P. cultellatus* |
| 65 | KP112376 | PCBH3 | *P. indicus* | Central China |  | *P. cultellatus* |
| 66 | KT951831 | PCBH3 | *P. indicus* | Qiongzhou Straits |  | *P. cultellatus* |
| 67 | JF952812 | P2SH1 | *P. indicus* | Japan |  | *Platycephalus* sp.2 |
| 68 | HQ711867 | PIRC | *P. indicus* | Rongcheng |  | *Platycephalus* sp.1 |
| 69 | KU236814 | PIRC | *P. indicus* | Rongcheng |  | *Platycephalus* sp.1 |
| 70 | HM180788 | P1SH1 | *P. indicus* | Korea |  | *Platycephalus* sp.1 |
| 71 | HM180789 | P1SH1 | *P. indicus* | Korea |  | *Platycephalus* sp.1 |
| 72 | HM180790 | P1SH1 | *P. indicus* | Korea |  | *Platycephalus* sp.1 |
| 73 | HM180791 | P1SH1 | *P. indicus* | Korea |  | *Platycephalus* sp.1 |
| 74 | HM180792 | P1SH1 | *P. indicus* | Korea |  | *Platycephalus* sp.1 |
| 75 | HM180793 | P1SH1 | *P. indicus* | Korea |  | *Platycephalus* sp.1 |
| 76 | HM180794 | P1SH1 | *P. indicus* | Korea |  | *Platycephalus* sp.1 |
| 77 | JQ738422 | P1SH4 | *P. indicus* | Yellow Sea |  | *Platycephalus* sp.1 |
| 78 | JQ738577 | P1SH1 | *P. indicus* | Yellow Sea |  | *Platycephalus* sp.1 |
| 79 | JQ738578 | P1SH1 | *P. indicus* | Yellow Sea |  | *Platycephalus* sp.1 |
| 80 | JQ738579 | P1SH1 | *P. indicus* | Yellow Sea |  | *Platycephalus* sp.1 |
| 81 | JQ738590 | P1SH1 | *P. indicus* | Yellow Sea |  | *Platycephalus* sp.1 |
| 82 | JQ738591 | P1SH1 | *P. indicus* | Yellow Sea |  | *Platycephalus* sp.1 |
| 83 | KP641486 | P1SH1 | *P. indicus* | Korea |  | *Platycephalus* sp.1 |
| 84 | KP641487 | P1SH1 | *P. indicus* | Korea |  | *Platycephalus* sp.1 |
| 85 | KP641488 | P1SH1 | *P. indicus* | Korea |  | *Platycephalus* sp.1 |
| 86 | KP641489 | P1SH4 | *P. indicus* | Korea |  | *Platycephalus* sp.1 |
| 87 | KP641490 | P1SH1 | *P. indicus* | Korea |  | *Platycephalus* sp.1 |
| 88 | KP641491 | P1SH4 | *P. indicus* | Korea |  | *Platycephalus* sp.1 |
| 89 | KP641492 | P1SH1 | *P. indicus* | Korea |  | *Platycephalus* sp.1 |
| 90 | KP641493 | P1SH1 | *P. indicus* | Korea |  | *Platycephalus* sp.1 |
| 91 | KU943397 | P1SH1 | *P. indicus* | Taiwan |  | *Platycephalus* sp.1 |
| 92 | KX147302 | P1SH1 | *P. indicus* | Vellar estuary |  | *Platycephalus* sp.1 |
| 93 | KX147303 | P1SH1 | *P. indicus* | Vellar estuary |  | *Platycephalus* sp.1 |
| 94 | KX147304 | P1SH1 | *P. indicus* | Vellar estuary |  | *Platycephalus* sp.1 |
| 95 | JX488287 | PCI2 | *P. cf. indicus* | Hongkong |  | Probably *Platycephalus* sp. |
| 96 | JX488145 | PCI1 | *P. cf. indicus* | Hongkong |  | Probably *Platycephalus* sp. |
| 97 | DQ107967 | PLA1 | *P. laevigatus* | Australia |  |  |
| 98 | DQ107980 | PLA1 | *P. laevigatus* | Australia |  |  |
| 99 | DQ107981 | PLA1 | *P. laevigatus* | Australia |  |  |
| 100 | JX488209 | PLA1 | *P. laevigatus* | Southeast Australia |  |  |
| 101 | JX488210 | PLA1 | *P. laevigatus* | Southeast Australia |  |  |
| 102 | JX488241 | PLA1 | *P. laevigatus* | South Australia |  |  |
| 103 | DQ107966 | PLA2 | *P. laevigatus* | Australia |  |  |
| 104 | DQ107968 | PLA2 | *P. laevigatus* | Australia |  |  |
| 105 | JX488206 | PLA2 | *P. laevigatus* | Southeast Australia |  |  |
| 106 | DQ107959 | PLO1 | *P. longispinis* | West Australia |  |  |
| 107 | DQ107971 | PLO1 | *P. longispinis* | Southeast Australia |  |  |
| 108 | DQ107969 | PLO2 | *P. longispinis* | Southeast Australia |  |  |
| 109 | DQ107970 | PLO2 | *P. longispinis* | Southeast Australia |  |  |
| 110 | DQ107960 | PLO3 | *P. longispinis* | West Australia |  |  |
| 111 | DQ107961 | PLO3 | *P. longispinis* | West Australia |  |  |
| 112 | JX488243 | PLO4 | *P. longispinis* | West Australia |  |  |
| 113 | JX488158 | PLO5 | *P. longispinis* | West Australia |  |  |
| 114 | DQ107972 | PLO6 | *P. longispinis* | Southeast Australia |  |  |
| 115 | DQ107973 | PLO7 | *P. longispinis* | Southeast Australia |  |  |
| 116 | DQ107962 | PLO8 | *P. longispinis* | West Australia |  |  |
| 117 | DQ107958 | PLO9 | *P. longispinis* | West Australia |  |  |
| 118 | DQ107951 | PM1 | *P. marmoratus* | Southeast Australia |  |  |
| 119 | DQ107963 | PM1 | *P. marmoratus* | Southeast Australia |  |  |
| 120 | DQ107965 | PM1 | *P. marmoratus* | Southeast Australia |  |  |
| 121 | DQ107950 | PM2 | *P. marmoratus* | Southeast Australia |  |  |
| 122 | JX488150 | PO1 | *P. orbitalis* | West Australia |  |  |
| 123 | JX488284 | PO1 | *P. orbitalis* | West Australia |  |  |
| 124 | JX488286 | PO2 | *P. orbitalis* | West Australia |  |  |
| 125 | DQ108006 | PR | *P. richardsoni* | Southeast Australia |  |  |
| 126 | JX488214 | PR | *P. richardsoni* | Southeast Australia |  |  |
| 127 | DQ107952 | PS1 | *P. speculator* | Southeast Australia |  |  |
| 128 | DQ107953 | PS1 | *P. speculator* | Southeast Australia |  |  |
| 129 | DQ107954 | PS1 | *P. speculator* | Southeast Australia |  |  |
| 130 | DQ107955 | PS1 | *P. speculator* | Southeast Australia |  |  |
| 131 | DQ107956 | PS1 | *P. speculator* | Southeast Australia |  |  |
| 132 | DQ107982 | PW1 | *P. westraliae* | West Australia |  |  |
| 133 | DQ107983 | PW1 | *P. westraliae* | West Australia |  |  |
| 134 | DQ107984 | PW1 | *P. westraliae* | Northeast Australia |  |  |
| 135 | DQ107985 | PW1 | *P. westraliae* | Southeast Australia |  |  |
| 136 | JX488141 | PW1 | *P. westraliae* | Northeast Australia |  |  |
| 137 | JX488172 | PW1 | *P. westraliae* | Northeast Australia |  |  |
| 138 | JX488192 | PW1 | *P. westraliae* | Northeast Australia |  |  |
| 139 | JX488217 | PW1 | *P. westraliae* | Northeast Australia |  |  |
| 140 | JX488238 | PW1 | *P. westraliae* | Northeast Australia |  |  |
| 141 | DQ107997 | PW2 | *P. westraliae* | West Australia |  |  |
| 142 | JX488187 | PW3 | *P. westraliae* | Northeast Australia |  |  |
| 143 | KU692768 | PV1 | *Platycephalus* sp.1 | Central Java |  | Not *Platycephalus* |
| 144 | KU692767 | PV2 | *Platycephalus* sp.1 | Central Java |  | Not *Platycephalus* |
| 145 | KU692766 | PV3 | *Platycephalus* sp.1 | Central Java |  | Not *Platycephalus* |
| 146 | JX488218 | PV4 | *P. indicus var 1* | Central Java |  | Clustered with *P. indicus* |
| 147 | JX488159 | PV5 | *P. indicus var 2* | East Java |  | Clustered with *P. indicus* of Okinawa |
| 148 | JX260936 | PV6 | *Platycephalus sp.* | Guangdong |  | Clustered with *P. indicus* |
| 149 | LC201784 | P2SH1 | *Platycephalus sp.* | Hiroshima Bay, Japan |  | *Platycephalus* sp.2 |
| 150 | EF607489 | P1SH1 | *Platycephalus sp.* | Guangdong |  | *Platycephalus* sp.1 |
| 151 | JX972197 | P1SH1 | *Platycephalus* sp.1 | China |  |  |
| 152 | JX972198 | P1SH1 | *Platycephalus* sp.1 | China |  |  |
| 153 | JX972199 | P1SH1 | *Platycephalus* sp.1 | China |  |  |
| 154 | JX972200 | P1SH1 | *Platycephalus* sp.1 | China |  |  |
| 155 | JX972201 | P1SH1 | *Platycephalus* sp.1 | China |  |  |
| 156 | JX972202 | P1SH1 | *Platycephalus* sp.1 | China |  |  |
| 157 | JX972203 | P1SH1 | *Platycephalus* sp.1 | China |  |  |
| 158 | JX972204 | P1SH1 | *Platycephalus* sp.1 | China |  |  |
| 159 | JX972206 | P1SH1 | *Platycephalus* sp.1 | China |  |  |
| 160 | JX972207 | P1SH1 | *Platycephalus* sp.1 | China |  |  |
| 161 | JX972208 | P1SH1 | *Platycephalus* sp.1 | China |  |  |
| 162 | JX972209 | P1SH1 | *Platycephalus* sp.1 | China |  |  |
| 163 | JX972211 | P1SH1 | *Platycephalus* sp.1 | China |  |  |
| 164 | JX972213 | P1SH1 | *Platycephalus* sp.1 | China |  |  |
| 165 | JX972215 | P1SH1 | *Platycephalus* sp.1 | China |  |  |
| 166 | JX972210 | P1SH2 | *Platycephalus* sp.1 | China |  |  |
| 167 | JX972205 | P1SH4 | *Platycephalus* sp.1 | China |  |  |
| 168 | JX972214 | P1SH4 | *Platycephalus* sp.1 | China |  |  |
