# Supplementary material for: Phylogeographic analysis of the genus Platycephalus along the coastline of the northwestern Pacific inferred by mitochondrial DNA
Source: BMC Evol Biol. 2019 Jul 31;19:159. doi: 10.1186/s12862-019-1477-1 (PMC6670200; doi:10.1186/s12862-019-1477-1)
Supplement: Supplementary file 5 — Table S3. Distribution of haplotypes shared among populations of the Platycephalus sp. 1. (DOCX 17 kb) [file 12862_2019_1477_MOESM5_ESM.docx]

Table S3. Distribution of haplotypes shared among populations of the *Platycephalus* sp. 1.

| Shared haplotype | No. of individuals | TO | DL | DY | WH | QD | NT | ZS | ND | CL | XM | ST | SZ | ZH | ZJ | BH | FC |
| --- | --- | --- | --- | --- | --- | --- | --- | --- | --- | --- | --- | --- | --- | --- | --- | --- | --- |
| SH1 | 156 |  | 19 | 16 | 10 | 22 | 3 | 18 | 4 | 19 | 6 |  | 1 |  | 6 | 18 | 14 |
| SH2 | 36 | 19 | 2 | 1 |  |  | 1 | 2 | 2 | 1 | 1 | 1 | 1 |  | 1 | 3 | 1 |
| SH3 | 34 |  | 6 | 3 |  | 3 | 1 | 1 | 1 | 2 | 1 |  |  |  | 2 | 6 | 8 |
| SH4 | 8 | 1 | 1 |  |  | 2 | 1 |  |  | 2 |  |  |  |  |  |  | 1 |
| SH5 | 8 |  |  | 3 |  | 2 |  | 1 |  |  |  | 1 |  | 1 |  |  |  |
| SH6 | 6 |  | 2 |  |  |  |  | 1 | 1 | 1 |  |  |  |  |  |  | 1 |
| SH7 | 6 |  |  |  |  |  | 1 | 2 | 1 |  |  |  |  |  |  |  | 2 |
| SH8 | 5 |  |  |  |  |  | 1 |  |  |  |  |  |  |  | 1 | 2 | 1 |
| SH9 | 5 |  |  |  |  |  |  | 1 | 1 | 1 |  |  |  |  | 1 |  | 1 |
| SH10 | 4 |  |  |  | 1 | 2 |  |  |  |  |  |  |  |  | 1 |  |  |
| SH11 | 3 |  |  | 1 |  |  |  | 1 |  |  |  |  |  |  |  | 1 |  |
| SH12 | 3 |  |  |  |  |  |  | 1 |  | 1 |  |  |  |  |  |  | 1 |
| SH13 | 3 |  |  |  |  |  |  |  | 1 |  |  |  |  |  |  | 2 |  |
| SH14 | 2 |  | 1 | 1 |  |  |  |  |  |  |  |  |  |  |  |  |  |
| SH15 | 2 |  |  |  |  | 1 |  | 1 |  |  |  |  |  |  |  |  |  |
| SH16 | 2 |  |  |  |  | 1 |  |  |  |  | 1 |  |  |  |  |  |  |
| SH17 | 2 |  |  |  |  |  | 1 |  | 1 |  |  |  |  |  |  |  |  |
| SH18 | 2 |  |  |  |  |  | 1 |  |  |  |  |  | 1 |  |  |  |  |
| SH19 | 2 |  |  |  |  |  |  | 1 |  | 1 |  |  |  |  |  |  |  |
| SH20 | 2 |  |  |  |  |  |  | 1 |  | 1 |  |  |  |  |  |  |  |
| SH21 | 2 |  |  |  |  |  |  |  |  |  | 1 |  |  |  |  | 1 |  |
| SH22 | 2 |  |  |  |  |  |  |  |  |  | 1 |  |  |  |  | 1 |  |
| SH23 | 2 |  |  |  |  |  |  |  |  |  | 1 |  |  |  |  | 1 |  |
| Total | 297 | 20 | 31 | 25 | 11 | 33 | 10 | 31 | 12 | 29 | 12 | 2 | 3 | 1 | 12 | 35 | 30 |
