# Supplementary material for: Phylogeographic analysis of the genus Platycephalus along the coastline of the northwestern Pacific inferred by mitochondrial DNA
Source: BMC Evol Biol. 2019 Jul 31;19:159. doi: 10.1186/s12862-019-1477-1 (PMC6670200; doi:10.1186/s12862-019-1477-1)
Supplement: Supplementary file 6 — Table S4. Comparison of selected morphological characters of four Platycephalus species from Chinese coastal seas and Pacific coast of Japan, as found in bibliography. (DOCX 20 kb) [file 12862_2019_1477_MOESM6_ESM.docx]

Table S4. Comparison of selected morphological characters of four Platycephalus species from Chinese coastal seas and Pacific coast of Japan, as found in bibliography.

|  |  | Records in references | | | |
| --- | --- | --- | --- | --- | --- |
|  |  | Qin et al., 2013 | Yasuji et al., 1996 | Chen et al., 2018 | Chen et al., 2017 |
|  |  | Platycephalus sp. 1 (n=174) | Platycephalus sp. 2 (n=15) | P. cultellatus  (n=48) | P. indicus  (n=13) |
| Measurements: | SL (mm) | 122.0-482.8 | 302-384 | 220-494 | 148.0-546.7 |
| Counts: | D1 | Ⅱ+Ⅵ-Ⅶ+0-Ⅰ (usually Ⅱ+Ⅶ+Ⅰ) | Ⅱ+Ⅶ+Ⅰ | Ⅰ-Ⅱ+Ⅶ+0-Ⅰ  (usually Ⅱ+Ⅶ+Ⅰ) | Ⅰ+Ⅶ+Ⅰ |
|  | D2 | 13-14 (usually 13) | 13 | 13-14 (usually 13) | 13 |
|  | A | 13-14 (usually 13) | 13 | 13 | 13 |
|  | P1 | 17-19 | 16-19 | 17-19 | 18-19 |
|  | C | 11-14 | - | 11-13 (usually 12 or 13) | 11-12 (usually 12) |
|  | LLS (spines) | 83-99 (1-2) | 67-74 | 65-76 | 73-80 (1-2) |
|  | OBS | 103-121 | 89-107 | 86-120 | 86-108 |
|  | GR | 11-17 (usually 14) | 11-13 | 7-10 (usually 9 or 10) | 6-9 |
|  |  |  |  |  |  |
| As% SL: | HL | 26.3-31.3 | - | 29.39-32.45 | 29.7-33.7 |
|  | PDL | 27.8-41.0 | - | 32.23-35.31 | 28.7-34.2 |
|  | LD1B | 14.0-22.1 | - | 13.28-18.63 | 16.2-22.3 |
|  | LD2B | 30.2-38.0 | - | 33.67-37.26 | 30.7-34.2 |
|  | LAB | 33.5-43.6 | - | 35.69-38.97 | 35.0-39.6 |
|  | SNL | 7.6-9.8 | - | 6.58-8.12 | 7.4-8.8 |
|  | OD | 3.1-9.9 | - | 3.6-4.83 | 3.2-6.7 |
|  | UJL | 9.8-14.3 | - | 8.19-9.82 | 10.9-12.8 |
|  | LJL | 13.5-19.9 | - | 8.39-11.28 | 14.9-17.6 |
|  | IW | 2.6-6.1 | - | 4.59-5.98 | 2.7-5.2 |
|  | POL | 15.9-22.3 | - | 18.28-21.41 | 16.8-19.4 |
|  | SW | 2.3-3.7 | - | - | 2.6-3.3 |
|  | P1L | 12.2-17.9 | - | 12.4-16.84 | 11.7-16.2 |
|  | P2L | 19.6-26.7 | - | 16.67-21.35 | 21.7-25.5 |
|  | CL | 14.1-20.8 | - | 13.23-16.38 | 15.0-19.7 |
|  |  |  |  |  |  |
| As% HL: | SNL | 23.8-31.3 | - | 21.65-25.25 | 24.5-29.7 |
|  | OD | 10.9-17.2 | 12.7-20.4 | 11.56-15.58 | 12.6-18.4 |
|  | UJL | 30.3-58.9 | - | 26.7-31.7 | 30.5-34.9 |
|  | LJL | 46.8-57.1 | - | 26.91-36.77 | 48.9-52.9 |
|  | IW | 9.8-19.6 | 9.2-17.9 | 15.07-18.5 | 22.7-28.0 |
|  | POL | 52.6-66.7 | 54.4-63.5 | 59.3-66.21 | 52.2-59.6 |
|  | SW | 6.7-11.2 | - | - | 6.9-9.6 |
| Caudal fin with (Y) or without (N) a yellow blotch | | N | N | N | Y |

D1, first dorsal-fin rays; D2, second dorsal-fin rays; A, anal-fin rays; P1, pectoral-fin rays; C, branched caudal-fin rays; LLS, pored lateral line scales; OBS, oblique body scale rows slanting downward and backward above lateral line; GR, gill rakers; TL, total length; SL, standard length; HL, head length; PDL, pre-dorsal length; LD1B, length of first dorsal-fin base; LD2B, length of second dorsal-fin base; LAB, length of anal-fin base; SNL, snout length; OD, orbital diameter; UJL, upper-jaw length; LJL,lower-jaw length; IW, interorbital width; POL, postorbital length; SW, suborbital width; P1L, pectoral-fin length; P2L, pelvic-fin length; CL, caudal-fin length.
