# Supplementary material for: Phylogeographic analysis of the genus Platycephalus along the coastline of the northwestern Pacific inferred by mitochondrial DNA
Source: BMC Evol Biol. 2019 Jul 31;19:159. doi: 10.1186/s12862-019-1477-1 (PMC6670200; doi:10.1186/s12862-019-1477-1)
Supplement: Supplementary file 7 — Table S5. Primers information of mitochondrial DNA for the Platycephalus. (DOCX 19 kb) [file 12862_2019_1477_MOESM7_ESM.docx]

Table S5. Primers information of mitochondrial DNA for the *Platycephalus.*

| Gene /Region | Primer name | Forward\Reserve | Primer sequence |
| --- | --- | --- | --- |
| *COI* | WYIF | Forward | CTCTCAGCCATCCTACCTG |
|  | WYIR | Reserve | AAAGAACCAGAAGAGGTGTTG |
| Cyt *b* | YBF | Forward | ACTCTAACCAGGACTAATGGCTTG |
|  | YBR | Reserve | ATGTAGGGGACTGCGGAGAG |
| Control region | DL-S | Forward | CCCACCACTAACTCCCAAAGC |
|  | YRR | Reserve | GCCCTGAAGTAGGAACCAAATG |
